# The active self in autism: multisensory and sensorimotor foundations of bodily self-representation

**DOI:** 10.1186/s12888-026-08756-w

**Published:** 2026-10-15

**Authors:** Amir Jahanian Najafabadi, Alireza Rastegari

**Affiliations:** Department of Cognitive Neuroscience, Bielefeld University, 33501 Bielefeld, Germany

**Keywords:** Autism spectrum disorder, Active self, Bodily representation, Embodied cognition, Multisensory integration, Sensorimotor processing, Self-other differentiation

## Abstract

The active self, understood as the embodied sense of self arising from goal-directed action and interaction with the environment, is supported by sensorimotor processes including multisensory integration and proprioception. Atypical self-representation in autism spectrum disorder (ASD) has been proposed to relate to differences in these processes. To clarify this relationship, we conducted a systematic conceptual review of 37 empirical studies published between 2010 and August 2025 examining multisensory and bodily components of the active self in ASD. The literature was synthesized across three themes: (1) multisensory integration, (2) self-bias and self-other differentiation, and (3) motor underpinnings of bodily awareness. Across studies, autistic participants frequently showed differences in multisensory timing and weighting, including reduced or delayed susceptibility to some bodily ownership illusions and greater reliance on proprioceptive or immediate sensory information. Evidence also indicated differences in the flexibility of peri- and interpersonal space, self-other differentiation, agency-related processing, and motor planning and execution. However, findings were not uniform across paradigms, developmental stages, or participant groups, with relatively preserved performance reported under some unimodal or low-noise conditions. Together, these findings support an association between multisensory and sensorimotor processing and several components of bodily self-representation in autism, while substantial methodological and sample heterogeneity limits strong causal or clinical conclusions. Future research should prioritize larger and more representative samples, longitudinal and multimethod designs, standardized experimental paradigms, and objective measures of embodiment to determine whether these mechanisms can ultimately inform individualized assessment and intervention.

**Clinical trial number **Not applicable.

## Introduction

Autism spectrum disorder (ASD) is a collection of disorders that affect a person’s ability to communicate and interact with others, resulting in difficulties in cognitive and emotional development [1]. This condition is characterized by aberrant development in interpersonal interaction, social bonding, and imaginative thinking. For example, insistence on doing the same things, stereotyped movements, rigid adherence to a routine, and difficulty establishing relationships, interests, and activities (Diagnostic and Statistical Manual of Mental Disorders, DSM-5-TR, 2022 [2]). These traits have been typically pinned on variances of mentalizing approaches and differences in associated neural networks [3–6].

The prevalence rate of ASD shows significant variation according to the geographical location, gender, participants’ age, and the date of conducting the surveillance. One of the most recent studies reported an overall pooled prevalence of 0.72% (which equals 72 per 10,000 for ASD [7]). However, according to the report from the Autism and Developmental Disabilities Monitoring (ADDM) Network, the prevalence rate can be as high as one in 36 eight-year-olds (about 2.7%) in the United States [8]. In addition to social problems, there has been an increased emphasis on diagnosing and comprehending individuals with ASD through the study of differences in sensory processing [9]. Over 90% of individuals with ASD have been shown to exhibit sensory differences, as evidenced by autistic individuals (e.g. [10, 11]). This underlines the role of sensory abnormalities as a defining feature in patients diagnosed with this neurodevelopmental disorder [12]. Autism has been called a condition of the self frequently [13, 14]. Interestingly, the term “autism” is derived from the Greek word autos, which means “self” [15]. Therefore, to comprehend the fundamental origin of autistic characteristics, it is essential to explore the concept of self, which lies at the core of the current study, particularly how self is constructed, represented, and experienced through bodily and multisensory processes in individuals diagnosed with autism.

ASD, as a heterogeneous neurodevelopmental disorder, is characterized by a wide range of cognitive, sensory, and motor profiles. One particularly complex domain is the experience of self-awareness, which can vary significantly across individuals on the spectrum. Self-awareness can be conceptualized across four levels: unconsciousness, consciousness, self-awareness, and meta-self-awareness, and individuals with ASD may exhibit distinct and individualized profiles at each of these levels [15, 16]. This variation is further compounded by impairment in sensory integration, a neurobiological process essential for coherent perception and interaction with the environment. Such impairments often manifest as hypersensitivity, sensory avoidance, diminished responsiveness, or sensory seeking behaviors and are linked to atypical temporal binding windows [12]. For instance, increased sensitivity to environmental noise and differences or difficulties in perception of auditory stimuli disrupt the integration of sensory information, thereby affecting how autistic individuals interpret and engage with their surroundings [17]. Beyond sensory processing, it has been stated that motor difficulties play a significant role in ASD. Inefficient motor control hinders the formation of effective links between physical actions and associated emotion, cognitive or social processes, further impacting the embodied sense of self [18].

Prior research discovered impairments in different aspects of movement control and coordination in autistic individuals. These alterations include poor coordination of limbs [19], difficulties with balance maintenance [20], impaired fine motor coordination [21], issues with grasp planning [22], and altered gait and posture control [23, 24]. Building on these studies, a conclusion can be reached that motor difficulties could potentially be considered a principal problem in individuals with ASD [25], also check [26, 27]). Further research reported that when children with ASD acquire a motor skill, the internal models they create establish a heightened connection between self-generated motor commands and proprioception [28]. The results indicated an increased reliance on cortical regions such as the primary motor cortex (M1) and somatosensory cortex, which encode movements in intrinsic (body-centered) coordinates. Conversely, a reduced reliance on regions like the premotor and posterior parietal cortices, which typically represent movements in extrinsic (environment-centered) coordinates [28].

Evidence regarding physical and embodied self-representation in ASD is mixed (for review [29]). Research in this area has primarily relied on tasks assessing self-recognition and agency, and has not converged on a consistent pattern of differences. For instance, an altered sense of agency in ASD has been reported by some studies [30], whereas others have found typical performance in action monitoring and action attribution [31], as well as an intact explicit sense of agency [32]. By contrast, evidence for impairments in the psychological, or self-evaluative, aspects of self-related cognition in ASD are comparatively more consistent. For instance, it has been proposed that alterations in Theory of Mind (ToM) may underlie many autistic traits, including difficulties in social interaction and communication. However, an alternative perspective argues that individuals with ASD may not exhibit a global deficit but rather experience selective impairments in specific domains of self-processing [33]. Taken together, these mixed findings on physical and embodied self-representation indicate that no clear consensus yet exists regarding the status of bodily-self components in ASD, nor the criteria by which autistic and typically developing individuals can be reliably distinguished in this domain. In the following sections, we examine these unresolved findings in greater depth, focusing on specific components of embodied self-representation in the autistic population.

### The concept of the self and its relation to body representation

The notion of the self, or selfhood, has intrigued philosophers since ancient times and continues to be a central focus in contemporary cognitive science, philosophy, and social psychology [34]. Modern approaches conceptualize the self not as a monolithic construct but as a dynamic, multidimensional framework. Among its core components, self-concept or self-image plays a pivotal role, involving how individuals perceive themselves and how they believe they are perceived by others. A comprehensive understanding of the self requires integrating internal self-perceptions with external, socially mediated perspectives. This includes accounting for both subjective experience and the influence of diverse social and psychological contexts that influence how others perceive things [35, 36].

Theoretical perspectives on the self vary widely and reflect ongoing debates across disciplines. Key points of these perspectives include: (I) Eliminativist accounts, which argue that the self is an illusion generated by neural processes and does not process ontological reality [37]; (II) Minimalist views, which consider the self to be a fleeting, moment to moment phenomenon tied to immediate experience and is rapidly and constantly changing [38–40]; (III) Narrative theories of the self, which posits that the self is purely formed by the stories individuals construct about their lives [41, 42]; (IV) Social constructivist approach, which suggest that the self is shaped by interpersonal and cultural influences, allowing for the existence of multiple, context-dependent selves [43]. Understanding the self through these diverse lenses sets the stage for investigating its embodied dimensions, particularly relevant when examining populations such as individuals diagnosed with ASD, where bodily self-representation and MSI may diverge from typical developmental trajectories.

At its most fundamental level, the concept of self is constructed on an individual’s bodily awareness, with particular emphasis placed on the face as the most important and uniquely salient and identity-defining physical attribute [44]. Gallagher described the active self, also referred to as the minimal self, as the immediate, pre-reflective sense of experience in relation to actions and perceptions in a specific situation [39]. This minimal self is particularly connected with the body part’s movement and its “sensorimotor grounding of self” which is linked to the perception of body ownership and sense of agency depending on one’s movement [39, 45]. The concept of the active self pertains to the interplay between perception, action, and prediction, forming a continuous yet flexible experiential framework through which one interprets and responds to the environment [46]. This concept is intricately linked to the phenomenological or experiential dimension of self-consciousness. Despite its transient nature, the minimal self maintains continuity and structure due to the innate structure of consciousness [47]. Crucially, the minimal self is an embodied entity, originating from basic proprioceptive (position sense) and kinesthetic (movement sense) processes that are sensitive and incorporate ecological information and bodily feedback [34]. The concepts of body ownership and sense of agency have proven instrumental in connecting body representations to bodily consciousness, offering insights into how individuals come to experience their body as their own [48]. Thus, the development and integration of body representations are essential prerequisites to the emergence of the minimal self, serving as a foundation for self-related experiences such as body ownership and sense of agency [49].

### Body representation: conceptual and clinical framework

Effective interaction with the environment and manipulation of our surroundings requires spatial, motor, and bodily representations. These representations contribute fundamentally to the conscious experience of oneself as an acting, embodied agent. To better conceptualize self-related phenomena, it is essential first to distinguish between the different types of body representation described in the literature.

**Fig. 1:**
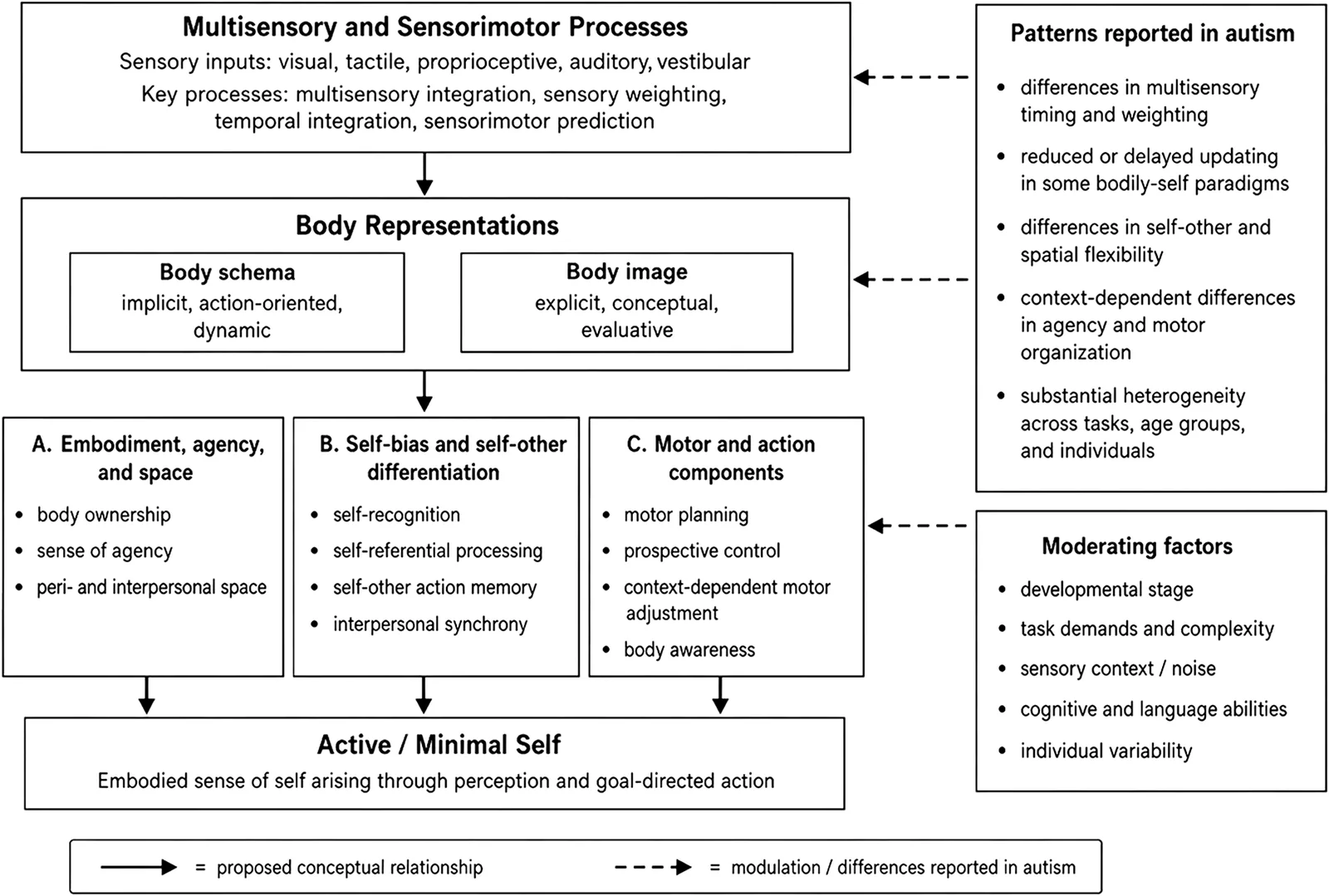
Conceptual synthesis of multisensory and sensorimotor contributions to the active self in autism. Multisensory and sensorimotor processes contribute to body representations, including body schema and body image, which interact with embodiment, agency, peri- and interpersonal space, self-other differentiation, and motor organization. Across these domains, the reviewed literature suggests differences in the timing, weighting, and flexibility with which sensory and contextual information is integrated and used to update bodily self-representations in autism, with substantial variation across developmental stages, task demands, and individuals. Arrows indicate proposed conceptual relationships and should not be interpreted as established causal pathways

Over the past two decades, multiple models have been proposed to classify types of body representation, yet a universally accepted taxonomy remains elusive due to the inherently multisensory and dynamic nature of body representation [50, 51]. According to Longo [51], body representation comprises at least six distinct types: body image, body schema, the superficial schema, the body model, as well as two aspects of somatic representation, which are semantic knowledge about the body and body structural description. However, this list is not exhaustive; the diversity of observed body-related deficits supports the need for even broader classifications [52]. Neuropsychological studies of both neurotypical and clinical populations offer support for these models speculated by Longo [51]. For example, some patients exhibit impairments in recognizing their own body parts or those of others [53]. Others experience distorted body size, such as patients with anorexia nervosa who overestimate their body dimensions despite being underweight [51, 54]. These clinical manifestations underscore the complexity and variability inherent in body representation. Expanding upon these findings, one widely accepted framework is the dyadic model of body representation [45, 55–57], which proposes that body representation, as a cognitive umbrella, comprises two core components of body image and body schema. This distinction lays the foundation for investigating how alterations in these subcomponents may relate to core symptoms of ASD and offers a valuable lens for understanding how MSI contributes to atypical self-experience in autistic individuals. Figure 1 illustrates the conceptual synthesis of multisensory and sensorimotor contributions to the active self in autism.

### Self-construction in autistic individuals

While the major focus of research on ASD traditionally examined the difficulties individuals face in understanding others, growing evidence suggests that self-referential processing, as the ability to process information related to the self, may also be atypical in individuals diagnosed with ASD. The evidence for atypical self-processing mechanisms controversially supports both hypotheses, strengthened self-bias and reduced self-referential processing. The hypothesis of heightened self-bias in autistic individuals stems from observations of extreme egocentrism (egocentric behaviors), which have been proposed as a core trait of ASD [58]. However, more recent studies challenge this view for autistic children and adults, indicating instead a decreased level of self-bias, i.e., inclination to selectively process personally relevant meaningful information [29, 35, 59, 60]. For instance, infants who exhibit a weaker reaction upon hearing their name have a greater possibility of getting diagnosed with ASD later in life [61]. Although this might be due to factors like delayed name acquisition or a weakened connection between a person’s name and social rewards [62], it can be used as a predictive value even before the age of one. The persistence of these differences in self-bias even in adulthood indicates a significant influence of impairment in sensory processing, particularly auditory processing, along with alteration of sensory integration in the autistic population [63]. These inconsistencies in outcomes such as reduced social rewards have led to the existence of different cognitive models aimed to elucidate the observed combination of typical and atypical self-referential processes in autistic individuals [62].

Evidence therefore does not support a simple distinction between an intact “physical self” and an impaired “psychological self” in autism. Instead, findings vary across components and levels of self-processing: relatively preserved performance has been reported for some basic self-recognition processes, whereas self-referential memory, self-other differentiation, and some agency-related measures show atypical patterns. This heterogeneity motivates examining individual components of the embodied self separately in the sections that follow [29, 35, 64].

### Sense of ownership in autism spectrum disorder

The concept of the bodily self has been extensively examined through various experimental paradigms such as the rubber hand illusion and full body illusion paradigms [65–67]. In parallel, researchers have utilized multisensory tasks to assess the immediate space surrounding the body, termed peripersonal space, using methods like audio-tactile and visuo-tactile stimulation [68–70]. These studies provide compelling evidence that the physical self is malleable and shaped by MSI processes arising from evidence of research focused on peripersonal space and full body illusions [71–73]. Fundamentally, the accurate integration of visual, tactile, and proprioceptive inputs underlies perception of our own body (i.e., body representation), including body localization (the ability to identify the location of one’s limbs) and body ownership as an intrinsic sense that our body belongs to us and can be seen, felt, and controlled [39, 74]. This sense of body ownership is crucial for developing motor competence and social interaction skills, both of which are frequently impaired in individuals with ASD. The problematic motor competence and social interactions of ASDs are in line with the observed altered sense of ownership in this population [75].

Considerable evidence supports the role of MSI impairment in the sensory and socio-communicative challenges associated with ASD [76–78]. Considerable evidence supports the role of differences in MSI in the sensory and socio-communicative challenges associated with ASD [76–78]. More recent findings further suggest that these differences may partly reflect prolonged temporal binding windows, particularly for visuo-tactile information. This extended temporal binding may contribute to differences in bodily self-representation in autistic individuals [79]. An additional account proposes that MSI in autism may also depend on attentional demands: autistic individuals may successfully integrate multisensory information under focused attention but show greater difficulty when attentional resources are divided [80].

The rubber hand illusion paradigm is a particularly useful paradigm to explore the sense of body ownership. In a classical version of this task, synchronous stroking of a participant’s hidden real hand and a visible fake hand leads to the illusion that the rubber hand belongs to them. Supporting this, a significant level of MSI in the formation of a sense of ownership over body parts can be seen in every specific incongruent condition in which conflicting visual, tactile, and proprioceptive stimulation lead to inaccuracies or illusions in perceiving ownership of a stimulus (i.e., rubber hand) [65] or a virtual body [66, 67], also check [81]. Rubber hand illusion tasks require detecting synchronous tactile stimulation with the visuo-tactile integration and the dominance of visuo-tactile synchrony over the proprioceptive information. This successful induction of the illusion depends on optimal visuo-tactile integration, which typically overrides conflicting proprioceptive input [82, 83]. Studies using the rubber hand illusion have reported differences in illusion susceptibility in autism, motivating closer examination of the multisensory mechanisms underlying body ownership [84]. This resistance cannot be explained and attributed solely to the general impairment of MSI.

As shown by Cowie et al. [85, 86], comparing the results of the rubber hand illusion in neurotypical children and adults suggests that two distinguished processes are responsible for the emergence of body ownership in the rubber hand illusion: One is a primary maturing system which is responsible for integrating visual and tactile cues widely addressed in existing models of body ownership [65, 82, 83], and another one is late-maturing system involved in self-localization, primarily relying on visual-proprioceptive integration. Proprioception is a sensory modality that provides information about the position and movement of one’s body and limbs, arising primarily from signals originating in muscles, tendons, and joints. In body ownership paradigms, proprioceptive estimates of limb position are integrated with visual and tactile information, allowing the nervous system to reconcile the seen and felt location of the body [87, 88].

During bodily illusions, visual-proprioceptive and visuo-tactile signals interact dynamically: visual and tactile synchrony can alter proprioceptive estimates of limb location, while the degree of visual-proprioceptive congruence constrains whether the multisensory evidence is incorporated into body representation [82, 83, 89, 90]. While both underlying mechanisms are operational by early childhood, the system supporting visual-proprioceptive localization continues to develop throughout late childhood [85, 86]. Thus, the extended visuo-tactile temporal binding window observed in autistic individuals may reflect delayed maturation of multisensory processes involved in body representation and may affect the development or updating of body ownership [79].

### Sense of agency in autism spectrum disorder

Both the sense of agency, described as the feeling that one is the initiator of one’s own actions, and the sense of ownership, as the feeling that one’s body or actions belong to oneself, are regarded as fundamental aspects of the bodily self [29]. The sense of agency, in particular, is a core component of human self-consciousness, allowing individuals to experience themselves as the agent behind their own voluntary movements [39]. A range of experimental paradigms has been employed to investigate the sense of agency across various theoretical frameworks, which has led to inconsistent findings. For instance, David et al. [32] found no impairment in sense of agency among autistic individuals using a visual feedback manipulation task designed to measure explicit sense of agency judgments. On the contrary, Grynszpan et al. [30] found diminished gaze stabilization in autistic individuals, interpreted as an impairment of gaze self-monitoring, possibly reflecting a diminished sense of agency [30].

In our opinion, it is possible to address these conflicting results through a more nuanced framework, such as the one proposed by Synofzik et al. [91]. In this framework [91], there are two distinguished aspects of sense of agency: judgment of agency and the feeling of agency. While the feeling of agency is a more implicit and pre-reflective experience, the judgment of agency is an explicit conceptual appraisal of agency. According to prior research (for review [92–94], these two parts of agency are affected differently when manipulated in the experimental tasks, suggesting that signals from motor and non-motor probably integrate differently at different levels of sense of agency and may involve distinct neural networks responsible for the interpretation of these diverse signals [32, 95]. Therefore, the aforementioned studies likely assessed different levels of sense of agency, potentially explaining their divergent findings. In line with this theoretical model and the discussion among the community, individuals with ASD are hypothesized to demonstrate a dissociation between the spared explicit process of sense of agency (i.e., judgment of agency), and the reduced or altered pre-reflective (implicit) aspect of sense of agency (i.e., feeling of agency) [96].

A complementary perspective concerns the motivational value of control. Beyond experiencing authorship over an action, being able to reliably control action outcomes may itself carry motivational value. From this perspective, alterations in agency in autism may involve not only differences in sensorimotor prediction or action monitoring but also differences in how action-effect contingencies contribute to the experience and motivational value of control. Recent findings showing atypical effects of action-effect delay on motor performance in autism [97], together with developmental evidence linking agency and reward in autism [98], suggest that motivational and sensorimotor accounts of agency may provide complementary explanatory levels.

Investigating the pre-reflective sense of agency is particularly challenging due to its implicit nature, but two well-established implicit experimental paradigms allow for indirect assessment: Intentional binding (IB) and sensory attenuation. Intentional binding refers to the subjective compression of the perceived time interval between a voluntary action and its sensory consequence, which has been considered a proxy for the feeling of agency, and sensory attenuation describes the reduced perception of intensity when a sensory consequence is self-generated, compared to when the same stimulus is produced by externally-generated action [99, 100]. It has been proposed by Waszak et al. [101] that within both measures, the pre-activation of the anticipated sensory consequence of the action can be observed. This pre-activation happens through the activation of the same neural regions during both measurements, including the dorsal-medial prefrontal cortex (dMPFC), cerebellum, supplementary motor cortex (SMA, which is known for its contributions to higher motor functions such as movement selection [102]), and anterior cingulate cortex (ACC).

Intentional binding is a major effect that has been investigated in many studies; it refers to the compression of the perceived temporal interval between a voluntary act and its consequence which is the demonstration of the feeling of agency over the act (for review [92–94, 99, 103–118]. Thus, observing diminished intentional binding effects in autistic people may be interpreted as an alteration in the pre-reflective or implicit aspect of sense of agency [96]. This diminished effect could be the result of the alteration of predictive mechanisms in ASDs (i.e., motor planning, action prediction, and monitoring) [119–126]. Such impairments support the hypothesis that the feeling of agency is particularly less flexible in ASD, even when explicit judgments of agency remain intact.

### Peripersonal space in autism spectrum disorder

Peripersonal space, as the immediate area surrounding the body where objects can be acted upon, is one of the critical aspects influenced by atypical sensory processing in ASD, particularly auditory and multisensory integration [127, 128]. Studies have shown conflicting findings regarding the size of peripersonal space in individuals with ASD: some report an expanded peripersonal space [129], while others suggest a reduced peripersonal space [130, 131]. Despite these discrepancies, a common finding across studies is the rigidity of peripersonal space boundaries in ASD. Unlike in neurotypical individuals, the peripersonal space does not adjust in response to social interactions [128]. This inflexibility reflects a steeper gradient between self and others, distinguishing ASD from conditions like schizophrenia, where this boundary is typically blurred.

Closely related to peripersonal space is interpersonal space (IPS), a construct from social psychology that refers to the distance individuals maintain from others to feel socially comfortable and emotionally secure [132, 133], specifically defines the distance we consider from other individuals to interact socially without experiencing discomfort and anxiety. While peripersonal space is associated with action potential, interpersonal space is more about social boundaries and the regulation of proximity during interpersonal interactions. Recent studies have extensively investigated the relation between these two spaces and the differences in their regulation and flexibility following tool use in ASDs and healthy individuals (for review [134–138]).

Recent research has examined how peripersonal space and interpersonal space are both modulated in ASD and typically developed, particularly in contexts involving tool use and social interaction [134–139]. These spatial representations have been investigated using different behavioral measures, including reaching-distance estimation to characterize perceived reachability or action space and comfort-distance tasks to assess interpersonal space. Importantly, reaching-distance estimation is not itself a direct measure of peripersonal space. Instead, it indexes the perceived limits of reachable, action-related space and is sensitive to changes in bodily action capabilities, including those induced by tool use [140–142]. Tool use can extend the functional possibilities of the body and consequently modify estimates of what can be reached, providing a useful behavioral index of action-related spatial plasticity. Although reachability space and peripersonal space are theoretically and experimentally distinguishable, both are body-centered, action-sensitive representations and may be jointly influenced by changes in body representation and sensorimotor experience. Moreover, the audio-tactile paradigm was also utilized to measure peripersonal space in some other studies [68, 143]. These studies presented synchronized sound and touch stimuli and assessed how the brain integrates sensory information to define the boundaries, offering valuable insights into peripersonal space, revealing how sensory integration defines the boundaries of the bodily self. In neurotypical individuals, both spaces are dynamic and can be flexibly altered. For example, using a long tool in a cooperative task with another person can expand peripersonal space and shrink interpersonal space due to enhanced engagement and social coordination [144–148]. The cooperative tool use paradigm results in the expansion of the reaching distance boundary, i.e., beyond the arm’s reach, possibly due to the interaction embedded within the task between the third person and participant (for review [145–148]).

Candini et al. [149] demonstrated that while the peripersonal space extended after tool use regardless of stimulus type (object or person) in autistic and neurotypical children, the interpersonal space regulatory mechanisms are different in ASD and typically developed individuals. Interpersonal space shrinks after collaborative tool use irrespective of tool size in the typically developed group, while the ASD group showed no significant changes in interpersonal space after the use of the tool, indicating that the regulation of interpersonal space was affected by autism, in contrast to the update of peripersonal space. This divergence implies that while sensorimotor updating of peripersonal space is preserved, the social flexibility of interpersonal space is less flexible. One explanation for this dissociation is that peripersonal space and interpersonal space, although related, may originate from distinct mechanisms. In this regard, peripersonal space originates from sensorimotor action potentials, and interpersonal space originates from affective and social cognitive processes. However, this separation is increasingly debated. Some studies argue for overlapping characteristics, including shared activation of frontoparietal brain regions and similar sensitivity to emotional and contextual variables [150–153]. These studies suggest that peripersonal and interpersonal space recruit partially overlapping frontoparietal networks [151, 152] and are both sensitive, although to different degrees, to social context, emotional state, and environmental properties in neurotypical individuals [134, 150, 153]. To fully understand the nuances of peripersonal space and interpersonal space in ASD, future studies should examine how factors like tool size, interaction quality (positive vs. negative), and movement agency (active vs. passive) influence these spatial representations. Such integrative research could clarify whether peripersonal space and interpersonal space arise from a common motor-social root or remain functionally and neurocognitively distinct in autistic individuals.

### Body image in autism spectrum disorder

Another essential aspect of body representation is body image, defined as the conscious awareness, perception, and evaluation of one’s own body [154]. Body image has been widely recognized as a significant psychological factor that influences an individual’s well-being and quality of life [155]. Broadly speaking, body image encompasses positive and negative perceptions, thoughts, emotions, behaviors, and attitudes related to one’s body and appearance (for review [156]). The development of body image is dynamic and shaped by physical characteristics (e.g., body size or shape), psychological variables (e.g., perfectionism, self-esteem), and sociocultural influences (e.g., media pressure, cultural ideals of beauty) [157, 158]. Importantly, body image is closely linked to the psychological experience of embodiment as the sense of owning, controlling, and being present within one’s body. Thus, body image does not merely reflect how individuals perceive their physical form; it also affects how they navigate the world through their bodily self [159].

Body image should be distinguished from the broader construct of embodiment. Whereas body image primarily concerns explicit perceptions, beliefs, and evaluations of one’s body, embodiment refers more broadly to the experience of inhabiting, owning, and acting through one’s body. These constructs are nevertheless related: multisensory and sensorimotor processes that establish a stable embodied representation may provide a foundation upon which more explicit evaluations of the body are constructed [160, 161]. Accordingly, alterations in multisensory integration, interoception, body ownership, or self-other distinction may indirectly influence body image in autism.

Recent studies have demonstrated strong associations between negative body image and autistic characteristics, suggesting that individuals with ASD or high autistic traits often experience atypical or distorted body image [162–164]. These findings suggest a possible reciprocal association: not only might altered embodiment and negative body image exacerbate social-communicative and sensory challenges in ASD, but the core features of ASD, such as diminished interoception, reduced self-other distinction, and altered MSI, could also contribute to difficulties in forming a coherent and stable body image. Taken together, investigating body image within the framework of embodiment provides valuable insights into the interconnectedness between bodily self-awareness and ASD traits, highlighting a critical but underexplored dimension of autistic experience.

### Current study

The present systematic conceptual review examines how multisensory and sensorimotor processes contribute to bodily self-representation and the active self in autism. Rather than treating the bodily self as a unitary construct, we synthesize evidence across three domains that organize the reviewed literature: (1) multisensory integration and bodily self-consciousness, including body ownership, sense of agency, and peri- and interpersonal space; (2) self-bias and self-other differentiation, including self-recognition and self-referential processing; and (3) motor function, action organization, and body awareness. We aim to identify where findings converge across paradigms, where they remain heterogeneous, and how differences in developmental stage, task characteristics, and sample composition may help explain divergent results. Specifically, we address three questions: (1) What patterns of multisensory and bodily self-processing are reported in autistic compared with non-autistic samples? (2) How do these patterns vary across developmental stages and experimental paradigms? (3) To what extent can the findings be integrated within an active-self framework linking multisensory processing, body representation, self-other differentiation, and action? We further identify methodological limitations and consider the implications of the available evidence for future mechanistic research, assessment, and intervention. The systematic search covered empirical studies published from 2010 through August 2025; the eligibility criteria, search strategy, screening procedure, and appraisal methods are described in the Methods.

## Methods

### Inclusion and exclusion criteria

To choose the most relevant articles in line with our systematic research, we included the original articles that were fully published in the English language starting from the year 2010 until August 2025. All measures had to be collected among the ASD population (with equal or more than 5 participants), across all ages, genders, nationalities, and the scope of interest. We included studies where the participants were primarily autistic individuals diagnosed by any method, whether or not they had other conditions. We did not include studies focused on other conditions that happened to have some autistic participants, which is not the focus of that study.

**Fig. 2:**
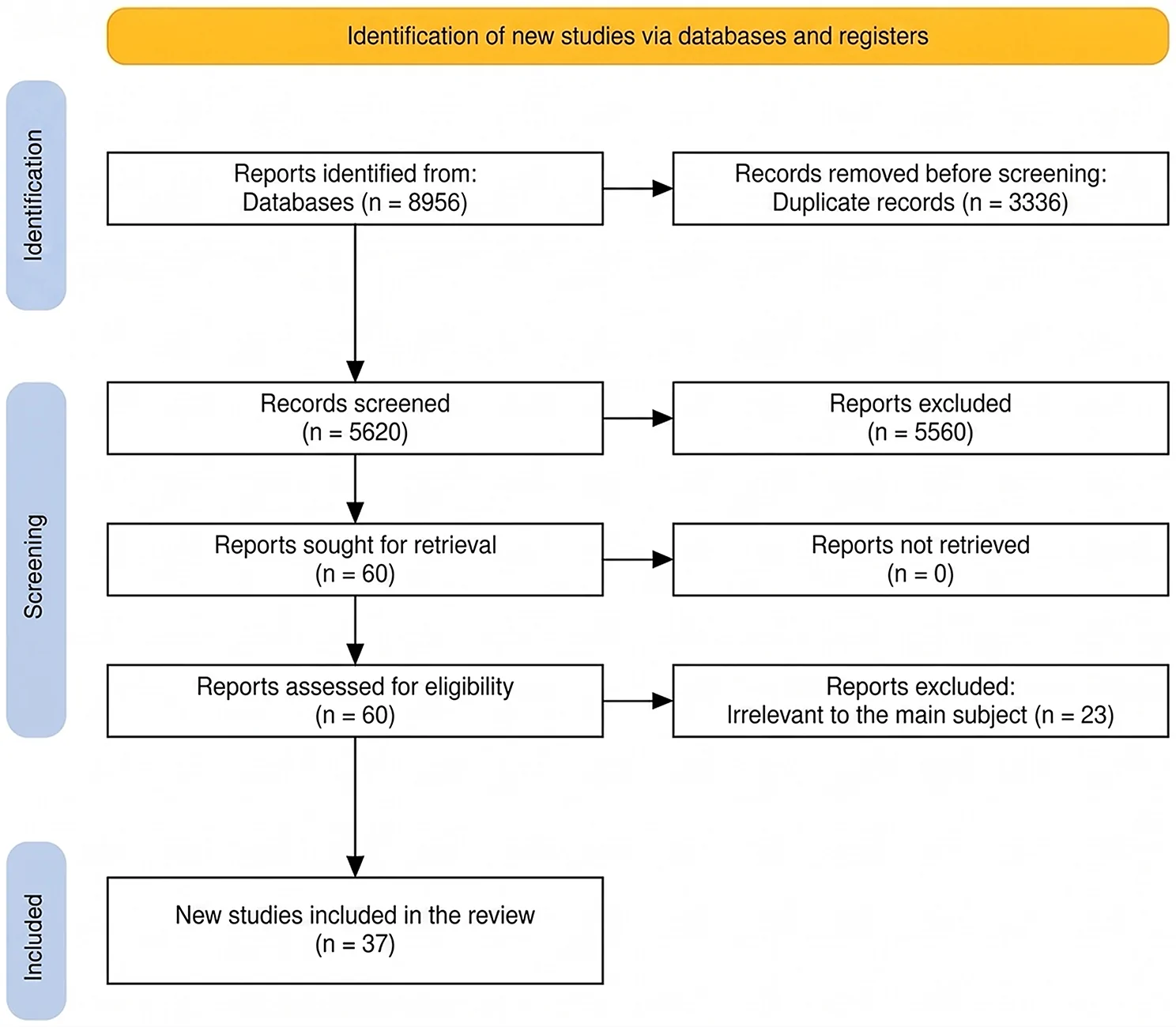
Preferred reporting items for systematic Reviews and meta-analyses (PRISMA) flow diagram

Quantitative and mixed-method empirical studies were eligible when they included quantitative or systematically assessed measures relevant to the review questions. The following exclusion criteria were used: studies that were conducted on animals; grey literature identified as abstracts, unpublished reports, and ongoing trials and reviews; and finally, non-empirical articles, including case reports, book chapters, letters, editorials, opinion pieces, and studies that included participants with additional physical or mental disorders, sensory disabilities, alcohol or drug abuse. This review was reported in accordance with the Preferred Reporting Items for Systematic Reviews and Meta-Analyses (PRISMA) statement [165], where applicable. The PRISMA flow diagram describing study identification, screening, eligibility assessment, and inclusion is presented in Fig. 2. The review protocol was not preregistered.

### Search strategy

Literature search was conducted in February 2024 and further updated in August 2025. The search for original articles was done in PubMed, Web of Science, ScienceDirect, Elsevier, and Scopus. Author AJN supervised and oversaw the search process. The final search combination has been brought into Box 1, with all the details. First, the results of the search were reported in the Endnote (version 20.6), but finally, for the ease of the process, it was decided to import them into the Rayyan web platform, which is designed for managing articles.

**Box 1:** Final search combination in PubMed, web of Science, ScienceDirect, Elsevier, and scopus

(autism OR autistic OR asperger OR asd OR hfa OR “high function”) **AND** (proprioceptive OR “visual-tactile” OR “multisensory integration” OR “visuo-tactile integration” OR “rubber hand illusion” OR rhi OR “sensory processing” OR “temporal binding window” OR “mental rotation” OR “perspective taking” OR “embodied interaction” OR “spatiotemporal representation” OR “space representation” OR “spatial perception” OR “body orientation” OR “temporal binding window” OR “visuo-tactile processing” OR “sensory perception” OR “cutaneous rabbit illusion” OR “proprioception” OR “dysfunctional mns” OR “visual self-body recognition” OR allocentric OR visuomotor OR “body awareness” OR “visuo-tactile stimulation” OR “embodied action” OR “sense of self” OR “minimal selfhood” OR “sense of presence” OR “self-perception” OR “multisensory integration” OR “self-other boundary” OR “bodily boundary” OR “self-other distinction” OR “self-controlled action” OR “self-agency monitoring” OR “self-agency” OR “agency monitoring” OR “comparator model” OR “intention in action” OR “judgments of agency” OR “explicit agency” OR joa OR “implicit agency” OR “feeling of agency” OR foa OR “intentional binding” OR “sensory attenuation” OR “visual-tactile spatial multisensory” OR “perceptual integration” OR “sensory processing abnormalities” OR “multisensory binding” OR “aberrant temporal binding of audiovisual stimuli” OR “pairing of audio and visual multisensory stimuli” OR “spatial multisensory integration” OR “cross-modal task” OR “multisensory temporal perception” OR extrapersonal OR interpersonal OR “atypical multisensory integration” OR “malleability of PPS” OR “multisensory integration” OR “plasticity of pps” OR “self-representation” OR “Neural self-representation” OR “minimalself” OR “activeself” OR “visual self-recognition” OR “physical self” OR “embodied self” OR “self-other discrimination” OR “self-reference effect” OR “self-related cognition” OR “evaluative self-related cognition” OR “self-related processing” OR “bodily experiences” OR “embodied mind” OR “bodily-based cognition” OR “perception of the bodily movements” OR “postural model of the body” OR “body position” OR “limb segments” OR “body segments” OR “body arrangement” OR “limb arrangement” OR “body configuration” OR “limb configuration” OR “body Spatial encoding” OR “body Spatial encoded” OR “body dimensional” OR “body dimension” OR “body spatial representation” OR “body organized model” OR “modular body” OR “adaptable body” OR “supramodal body” OR “coherent body” OR “body surface” OR “extended body” OR “body conception” OR “deafferentation” OR “the spatial unity of the body” OR “autotopagnosia” OR “phantom limb” OR “short term plasticity of the body schema” OR “somatoparaphrenia” OR “unilateral neglect” OR “alice in wonderland syndrome” OR “anorexia nervosa” OR “bulimia nervosa” OR “binge eating disorder” OR “body dysmorphic disorder” OR “body integrity dysphoria” OR “cotard delusion” OR “body size” OR “body dissatisfaction” OR “Self-location” OR “synchronous visual and tactile stimulation” OR “self-identification” OR “identified-self” OR “bodily self-experiences” OR “first person-perspective” OR “body integrity dysphoria” OR “body location” OR “representation of the bodily self” OR “muscle dysmorphia” OR “eating disorder” OR “body shape perception” OR “binge eating behaviours” OR “bodily changes” OR “weak central coherence” OR “bodily behaviours” OR “drive for muscularity” OR “the eating attitudes test” OR “maladaptive eating” OR “asomatognosia” OR “somatoparaphrenia” OR “anosognosia” OR “anosodiaphoria” OR “allochiria” OR “hemispatial neglect” OR “Body maps” OR “neural body representations” OR “somatotopic maps of the body” OR “offline representations” OR “online representations” OR “body model” OR “body structural description” OR “superficial schema” OR “audio-tactile integration” OR “visual-tactile integration” OR “asynchronous sensory” OR “temporally audio-visual binding” OR “atypical audio-visual binding” OR “extended temporal binding” OR “body metric representation” OR “boundaries of body”).	-

### Screening of references and eligibility assessment

Authors AJN and AR performed the screening process independently. By searching the above-mentioned databases, around 8956 were collected, and after removing the duplicates, 5620 papers were left for further steps. These articles were divided between authors randomly by checking the titles and the abstracts, and after negotiating about the doubtful articles, the final ones were chosen based on the criteria mentioned above. Finally, 37 articles were included. Following full-text eligibility assessment, data were extracted using a standardized extraction form developed for the present review.

### Data extraction and study variables

For the process of data extraction, the included studies were divided randomly between AJN and AR. Each reference’s data was extracted and imported to Microsoft Excel to discuss, finalize, and chart the data extracted. Included data brought in Excel sheets as follows authors, year, title, design/method (Study type), document type, significant level & effect size, the aim of the study, main group, measure and tools used for comparison group/s and main group, tasks obtained for comparison group/s and main group, outcomes for comparison group/s and main group, conclusion, suggestions and limitations of the study.

### Risk of bias assessment

**Fig. 3:**
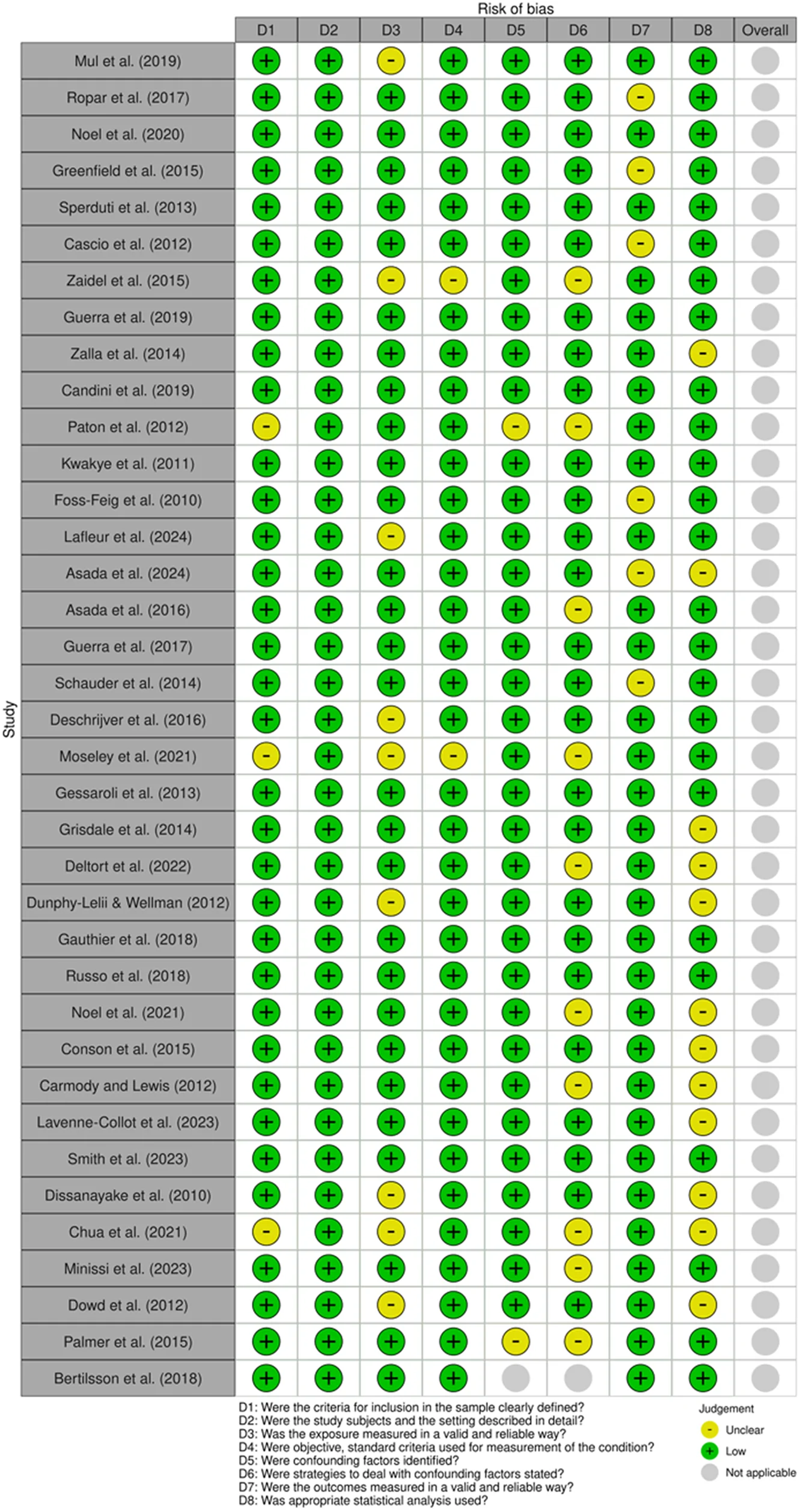
Item-level methodological quality and risk-of-bias appraisal of the included studies. Judgments were based on the 2020 JBI critical appraisal checklist for analytical cross-sectional studies [166]. Green indicates that the criterion was met (low concern), yellow indicates an unclear judgment because of insufficient information, red indicates that the criterion was not met (high concern), and grey indicates not applicable. No overall study-level score was calculated

Risk of bias and methodological quality were assessed independently by AJN and AR using the 2020 JBI Critical Appraisal Checklist for Analytical Cross-Sectional Studies [166]. This checklist was selected as a common appraisal framework because the majority of the included studies employed cross-sectional group-comparison designs. The checklist comprises eight items assessing: (1) clarity of the sample inclusion criteria; (2) adequacy of the description of participants and setting; (3) validity and reliability of exposure measurement; (4) use of objective and standardized criteria for measurement of the condition; (5) identification of potential confounding factors; (6) strategies for addressing confounding factors; (7) validity and reliability of outcome measurement; and (8) appropriateness of the statistical analysis. Each item was judged according to whether the criterion was met, not met, unclear because of insufficient information, or not applicable. For visualization in Risk-of-bias visualization (robvis [167]), these judgments were represented as low concern, high concern, unclear, or not applicable, respectively. No overall study-level score was calculated because no prespecified rule was used to aggregate the eight individual items. Disagreements between the two reviewers were resolved through discussion and consensus, with consultation of a third researcher where necessary. The item-level judgments for the included studies are presented in Fig. 3.

## Results

### Characteristics of included studies and participants

Across the 37 included studies, participant samples spanned a broad developmental range, from preschool-aged children to adults. Approximately 18 studies focused primarily on children and/or adolescents, 18 examined adult samples, and one employed a longitudinal design examining developmental change over time. Among studies reporting explicit age ranges, the youngest participants were approximately 3 years old, although the upper age limit was not consistently reported across the adult studies. The samples were also strongly male-skewed, with males comprising the majority of autistic participants in most studies that reported sex and several studies including exclusively male samples. Seven studies explicitly recruited participants described as having high-functioning autism and/or Asperger syndrome, further indicating that the available evidence is weighted toward autistic individuals without substantial cognitive impairment. Representation of autistic individuals with intellectual disability was very limited; in some cases, intellectual disability was explicitly absent from the autistic sample or was represented only through a separate comparison group. Likewise, none of the included studies explicitly identified minimally verbal or non-verbal autistic participants as a distinct sample, limiting the generalizability of the findings across the full spectrum of autistic individuals. Diagnostic ascertainment varied across studies, consistent with the broad inclusion criterion allowing autism diagnoses established using different methods. Where reported, standardized measures used for diagnostic confirmation or clinical characterization included the Autism Diagnostic Observation Schedule (ADOS) and Social Communication Questionnaire (SCQ), while measures such as the Autism-Spectrum Quotient (AQ) were also used to characterize autistic traits. Because IQ and verbal ability were not reported consistently across studies, a participant-level prevalence of higher versus lower cognitive/verbal functioning could not be estimated without imposing retrospective classifications not used by the original studies. The available literature should therefore be interpreted as disproportionately representing verbally able autistic individuals and as providing limited evidence concerning individuals with intellectual disability or minimal verbal ability.

### Multisensory integration and bodily self-consciousness in autism spectrum disorder

Across the reviewed literature, several recurring but non-uniform patterns emerged in multisensory integration, bodily self-consciousness, spatial regulation, and agency-related processing in autistic compared with typically developing participants. Studies of body ownership illusions revealed that ASD groups were less susceptible or displayed delayed responses to the full body illusion [168], numbness illusion [75, 169], and rubber hand illusion [84, 170–172]. These alterations were often accompanied by reduced proprioceptive drift [84], delayed visuo-tactile integration [170], or diminished self-identification with a virtual body [168]. Findings point to a more rigid self-other distinction and greater reliance on proprioceptive cues in ASD.

Multiple studies demonstrated extended visuo-tactile and audio-visual temporal binding windows in ASD, indicating atypical multisensory timing. This was shown in visuo-tactile illusion paradigms [79, 173], audio-visual temporal judgment tasks [174, 175], and flash-beep illusions [175]. These differences suggest delayed development of MSI processes that normally refine body representation and perceptual coherence.

Research on peripersonal and interpersonal space found ASD participants to have smaller and less flexible boundaries. Studies using peripersonal space paradigms reported sharper and more restricted peripersonal space gradients in ASD [168, 176]. Interpersonal space studies reported reduced preferred distances from others and less plasticity across social contexts [149, 177, 178]. This contrasts with typically developed individuals, who flexibly adjusted social distances depending on eye contact, cooperative contexts, and developmental stage.

With respect to interoception, Schauder et al. [171] found that ASD children showed stable heartbeat detection performance across intervals and reduced illusion susceptibility with higher interoceptive awareness, reinforcing the proposed role of interoception in shaping body ownership.

Sense of agency was also found to be altered. Reduced intentional binding effects were observed in ASD compared to typically developed individuals [96, 179], with diminished reliance on sensorimotor cues for agency judgments [180]. These atypicalities were further linked to reduced mentalizing performance in Theory of Mind tasks [180], suggesting an association between sensorimotor agency-related processing and higher-order social cognition.

Finally, in the context of self-motion and multisensory integration, Zaidel et al. [172] showed that while ASD participants performed similarly or better than typically developed individuals under noiseless or unimodal conditions, they were disproportionately affected by visual noise and relied more heavily on immediate sensory input than on prior knowledge. Collectively, these findings across paradigms [75, 79, 84, 96, 149, 168–180]. Taken together, these studies indicate recurring differences in multisensory timing and weighting, susceptibility to some bodily illusions, spatial representations surrounding the body, and agency-related processing, although the direction and magnitude of these effects vary across paradigms and participant groups. These atypicalities may contribute to broader difficulties in self-representation and social interaction. For detailed reviewed articles see Table 1.

**Table 1:** Summary of empirical studies investigating bodily self-consciousness, body ownership, interoception, peripersonal/interpersonal space, multisensory integration, and sense of agency in individuals with ASD compared to typically developing and other control groups. The table outlines study aims, participant groups, experimental tasks, and key findings

Study summary			Aim of Study	Target group		Tasks	Results
NO	Authors	Title		Main group	Comparison Group/s		
1	Mul et al. [168]	Altered bodily self-consciousness and peri-personal space in autism	Examining whether impaired multisensory processes in individuals with ASD could result in alteration of bodily self-consciousness	22 ASD adults (*M* = 14, F = 8; age Mean = 27.0 years, SD = 9.0)	29 TDs (age Mean = 27.2 years, SD = 6.7)	Full body illusion (FBI)	Compared to TDs, ASDs exhibited smaller peripersonal space with a steeper gradient at its boundary, and they were noticeably less susceptible to FBI than the control group, which suggests a more pronounced self-other distinction in ASDs
						Audio-tactile reaction time task	
						FBI self-identification	Opposite to ASDs, TDs showed greater self-identification with the virtual body and greater self-location drift towards the virtual body in the synchronous condition
						Multidimensional Assessment of Interoceptive Awareness	
							ASDs scored significantly different on the autistic traits (AQ), empathy (QCAE), and cognitive empathy (QCAEcog) questionnaire than TDs
						Audio-tactile integration task (Peripersonal space measurement)	
2	Guerra et al. [75]	In sync or not in sync? Illusory body ownership in ASD	Investigating the behavioral indications of the numbness illusion (NI) in individuals with ASD, to assess the alteration of their body ownership experiences as their finger got stroked by themselves and by another agent in synchronous and asynchronous condition	41 ASDs (*M* = 30, F = 11; age Mean = 24.78 years, SD = 3.37; range = 19–31 years)	41 TDs (*M* = 30, F = 11; age Mean = 24.56 years, SD = 4.35; range = 19–36 years)	Numbness Illusionary (NI)	In both groups, the NI was more evident for the ‘Self’ than for the ‘Other’ conditions, suggesting self-stroking corresponds with stronger interruption of tactile sensation and finger ownership. This suggests that modulation of the perceptual impact of touch is partially typical in ASDs
							In the self-stoking trials, TDs, opposite to ASDs, perceived the NI only if the stroking was performed asynchronously.
							No significant differences between ‘Other-stroking’ configurations within and between groups (ASDs vs TDs)
3	Ropar et al. [79]	Body representation difficulties in children and adolescents with autism may be due to delayed development of visuo-tactile temporal binding	Assenting the possible temporal extension of visuo-tactile integration as underlying cause of body representation difficulty	29 ASDs children/adolescents (age Mean = 151.65 months, SD = 23.07; range = 99.72–191.04 months)	28 CA-matched TDs (*M* = 20,F = 8; (age Mean = 152.18 months, SD = 19.85; range = 116.76–184 months)	RHI task with a multisensory illusion apparatus (MIRAGE)	Only ASDs and MA-matched TDs showed a reduced embodiment of the spatially incongruent and temporally congruent hand at shorter temporal delays. This suggests the possibility of a delay in visuo-tactile integration development in individuals with ASD
							In the spatially incongruent condition, only the CA group demonstrated significantly reduced accuracy across all three delays (60, 300, 180). However, for the MA group, scores were significantly lower than 100 only in the spatially incongruent 180 MS condition, and not in the other two delays. In terms of spatially congruent conditions, the CA group scores were only significantly lower when the delay was 60 Ms.
					27 Verbal-MA-matched TDs (*M* = 17, F = 10, (age *M* = 95.29 months, SD = 16.99; range = 64.00–123.6 months)	Social Aptitude Scale (SAS)	A significant but small positive correlation was found between average congruency scores and the scores of the Social Aptitude Scale (SAS). Indicating that those with better social skills showed a higher embodiment of the spatially incongruent hand.
4	Noel et al. [176]	Visual-Tactile Spatial Multisensory Interaction in Adults with Autism and Schizophrenia	Exploring the alteration of multisensory processing and social difficulties in ASD and schizophrenia patients aged between 18 and 60 years old Exploring the alteration of multisensory processing and social difficulties in ASD and schizophrenia patients aged between 18 and 60 years old Exploring the alteration of multisensory processing and social difficulties in ASD and schizophrenia patients aged between 18 and 60 years old	26 ASD adults (*M* = 14, F = 12; age Mean = 25.65 years, SD = 6.05; age range = 18–60 years)	36 TDs (*M* = 23, F = 13; age Mean = 33.56 years, SD = 11.19; age range = 18–60 years)	Cross-modal congruency task in 2 conditions (spatially congruent & incongruent)	All three groups (ASDs, SZ, TDs) showed the same performance regarding the cross-modal congruency task. Also, all groups’ reaction time responses were indicative of the facilitation of tactile processing when multisensory trials were executed at the nearest distance
				22 schizophrenics (SZ), adults (Mean = 13, F = 8, gender unknown = 1; age Mean = 45.09 years, SD = 9.94; age range = 18–60 years)		Reaction time (reaction time of the participants to touch in 2 conditions [with or without the presence of the dynamic visual stimuli which appeared to approach or withdraw from the body, within a PPS distance around the body])	The peripersonal space was smaller and more defined in the ASD group compared to SZ and TDs. In all groups, small peripersonal size was significantly correlated to the severity of impaired social symptoms, this correlation was largely driven by the TD group and had no statistically meaningful effects on either clinical group (ASD and SZ).
							Regarding evaluating the influences on the cross-modal congruency effect as defined by reaction time, the five predictive factors (diagnostic group, age, gender, IQ, and tactile-only RTs) could not forecast the multisensory congruency effect
5	Schauder et al. [171]	Interoceptive ability and body awareness in ASD	Measuring the interoceptive ability (detection, attention, and overall awareness) and investigating the possible association between interoceptive awareness (IA) and susceptibility to the RHI in neurotypical and autistic children	21ASDs children (*M* = 19, F = 2; age Mean = 12.28 years, SD = 2.8, age range = 8–17 years)	24 TDs children (*M* = 20, F = 4; age Mean = 11.52 years, SD = 2.5)	Heartbeat perception paradigm (to measure interoceptive ability)	A significant main effect of the interval was found in all the intervals (25, 35, 45, and 100 s), indicating that as the time interval became more extended, the performance became poorer.
						RHI task (15 of the 40 participants)	
							Compared to TDs, ASDs showed a more stable pattern across the intervals.
			Investigating differences in perception and devoting attention to internal and external cues and the alteration of body perception in children with ASD.			Visual counting control task	Higher Interoceptive awareness was associated with the reduction of RHI susceptibility. While Interoceptive awareness accounted for 16% of the variance in rubber hand drift, group and the interaction of group and Interoceptive awareness didn’t uniquely account for any significant drift in RHI
6	Greenfield et al. [173]	Visuo-tactile integration in autism: atypical temporal binding may underlie greater reliance on proprioceptive information	Obtaining conclusive evidence for the temporal extension of visuo-tactile binding in autistic individuals	29 ASDs children ((final analyzed sample; age *M* = 151.65 months, SD = 23.07; range = 99.72–191.04 months)	29 CA-matched TDs (*M* = 2,F = 8; age Mean = 146.13 months, SD = 21.35; range = 101.0–184 months)	Multisensory illusion apparatus (seeing two identical live video images of their own hand, one of them was aligned proprioceptively with the actual hand [the veridical hand], participants were asked to identify the image of the real hand while seeing the brush stoke by three different temporal delays which was applied to either the displaced hand or the veridical hand)	In all 3 groups, the synchronous hand was chosen more frequently in the congruent and the longer temporal delay conditions.
							Compared to ASDs, both TD groups (CA and MA) chose the synchronous hand more often
					29 Verbal-MA-matched TDs (*M* = 19, F = 10, age Mean = 94.56 months, SD = 16.68; range = 63.48–123.6 months)		Opposite to TDs, ASDs only consistently choose the synchronous hand in the congruent 180- and 300-ms conditions (not consistent in the congruent 60-ms condition). This suggests that short delay detection is difficult for ASDs.
							CA-matched TDs chose the synchronous hand significantly more often than the ASDs. In the congruent 60 ms condition, while the MA-matched TDs and ASDs had the same performance
							Opposite to ASDs, TDs were led by visuo-tactile temporal synchrony, even if this information was in contrast with proprioceptive information.
7	Sperduti et al. [96]	Altered Pre-reflective Sense of Agency in ASDs as Revealed by Reduced Intentional Binding	exploring the pre-reflective level of Sense of Agency (SoA) and the possible alteration of the implicit process of SoA by using Intentional Binding (IB) in individuals with Autism	15 male adults with ASD (AS, HFA, or PDD-NOS; *M* = 15, F = 0; age Mean = 33.53 years, SD = 11.01)	17 CA-matched TDs (*M* = 17, F = 0; age Mean = 33.06 years, SD = 11.13)	Interval timing procedure in two different conditions (control vs operant condition) for assessing intentional binding. Three different kinds of sensory stimuli (visual, auditory, and multimodal) after a variable temporal delay (250, 450, 650 ms. For the interpretation of the gathered results after the IB task, the more positive the proportion difference score (PDS), the greater the IB effect.	There was no significant difference in the ASDs, between the two conditions (OP and CO), for all intervals, when presented with visual stimuli
							A significant main effect of the group was found resulting from a greater PDS (=greater intentional binding) in TD compared to ASDs
							PDS was significantly different from zero in the TD group for all conditions, which shows the IB effect in all conditions for TDs
							General underestimation of the interval duration in the following order: Visual > Auditory > Multimodal
							A main effect of interval duration was found due to more underestimation of the intermediate and the longest intervals compared to the shortest one, while there were no differences between the two longer intervals.
8	Cascio et al. [170]	The rubber hand illusion in children with ASDs: delayed influence o combined tactile and visual input on proprioception	Comparing the TDs and ASDs in the susceptibility rate to RHI and exploring the multisensory integration or interhemispheric transfer as the plausible underlying factor	21 ASDs Children (76% male; age 8–17 years, Mean = 11.9, SD = 2.8 years)	28 TDs Children (93% male; age 8–17 years, *M* = 13.4, SD = 2.7 years)	RHI task	ASDs showed delayed susceptibility to RHI compared to typically developing children.
							ASDs exhibited similar susceptibility to the illusion across conditions following the first brushing block. However, ASDs showed an increase in drift in the synchronous condition, opposite to the asynchronous condition.
							Regarding the subjective report of the illusion, ASDs, opposite to TDs, were less likely to negatively endorse the item “It seemed as though the touch I felt was caused by the brush touching the rubber hand” in the asynchronous condition.
9	Zaidel et al. [172]	Self-motion perception in autism is compromised by visual noise but integrated optimally across multiple senses	Investigation of the possible reduction in multisensory integration and/or attenuated Bayesian priors in ASDs through the self-motion perception task	14 ASDs (*M* = 14, F = 0; age range = 13–19 years)	22 age-matched TD adolescents (*M* = 22, F = 0; age range = 13–19 years)	Self-motion perception task: presenting stimuli that simulated self-motion through a random dot cloud (star-field) and asking the participants to indicate the direction of perceived self-motion. The stimuli were presented under different conditions, including vestibular-only, visual-only, and combined vestibular and visual cues.	ASDs showed a high sensitivity to visual sensory noise. However, their performance in noiseless visual (100% coherence), vestibular, and multisensory integration conditions was no worse (and often even better) than TDs.
							ASDs showed a stronger reliance on incoming sensory information and less use of prior knowledge about the world (i.e., attenuated Bayesian priors).
10	Guerra et al. [169]	Sex Differences in Body Ownership in Adults with ASD	Exploring the effect of sex on the feeling of body ownership in TDs and ASDs	39 HFA ASDs (*M* = 29, F = 10; age Mean = 24.72 years, SD = 3.37; range = 19–31 years)	69 TDs (*M* = 43, F = 26; age Mean = 23.64 years, SD = 3.10; range = 19–33 years)	RHI task	ASDs showed a different pattern of results, with a weaker or absent illusion in both conditions (synchronous vs asynchronous).
			Investigating the possible relation between the feeling of ownership and empathy in ASDs			Numbness Illusion (NI) task	While synchronous self-stroking produced the illusory effect of the NI in both TDs and ASDs, TDs didn’t perceive the illusion in the asynchronous condition.
11	Zalla et al. [180]	Metacognition of agency and theory of mind in adults with high functioning autism	Exploring the possible alteration of the metacognition of sense of agency in HFAs and its potential relation with the impairments in Theory of Mind (ToM) in the autistic group through the cue-integration task	19 HFAs (*M* = 16, F = 3; age Mean = 28.8 years, SD = 7.1; range = 20–45 years)	19 CA-matched TDs (*M* = 15, F = 4; age Mean = 26.4 years, SD = 6.1; range = 20–43 years)	A computer task: for manipulation of the sense of agency by distorting the objective control, either by adding random noise or time lag; to assess the participants’ sensitivity to discrepancies in cues	Compared to TDs, HFAs showed a diminished use of the diagnostic sensori-motor cues in making metacognitive judgments of agency, and accurate metacognitive judgments of performance.
							Independent of the experimental condition, HFAs reported a higher judgment of agency and judgment of performance than TDs
						Faux-pas Recognition Task (for assessing theory of mind)	Both TDs and HFAs made their judgments of agency based on the external cues (judgements of performance). However, HFAs showed an under reliance on the internal diagnostic agency cues generated during the experimental manipulations
							In HFAs, the degree of the under reliance on internal sensory-motor cues correlated with poor performance on the Faux Pas task, which shows the low level of mentalizing abilities.
12	Candini et al. [149]	When social and action spaces diverge: A study in children with typical development and autism	Investigation of the functional properties and the degree of the plasticity of the peripersonal space and interpersonal space with in the ASDs and TDs community by using cooperative tool-use paradigm	Exp 1: 18 HFA children (age Mean = 10.9 years, SD = 1.64)	Exp 1: 18 MA-matched TDs (age Mean = 11.1 years, SD = 1.83)	Reaching-distance task	Expansion of the peripersonal space following tool use with the long tool, opposite of the short tool, in both TDs and ASDs
						comfort-distance task	In TDs, opposite to ASDs, the interpersonal space toward confederate reduced after cooperative tool-use training
				Exp 2: 18 HFA children (age *M* = 11.2 years, SD = 1.72)	Exp 2: 18 MA-matched TDs (age *M* = 11.2 years, SD = 2.28)		
						Cooperative tool-use training	Results were suggestive of the intact action space and altered social space within ASDs
13	Paton et al. [84]	The Rubber Hand Illusion Reveals Proprioceptive and Sensorimotor Differences in ASDs	Exploring the possible difference in unimodal vs multi-modal sensory processing and the preference of the local over global processing in ASDs through the modified versions of RHI	17 HFA (*M* = 14, F = 3; age *M* = 32.06 years, SD = 12.43)	17 TDs (*M* = 12, F = 5; age *M* = 27.06 years, SD = 6.20)	Modified version of RHI (4 within-subject factors: Synchrony, Goggles (Goggles or No Goggles), Item (Rubber hand or Cardboard Box), Stimulation (Manual Touch or Machine Touch)	RHI occurred in both TDs and ASDs
							Comparing to TDs, ASDs showed more accurate proprioception and reduction in sensitivity towards visuo-tactile proprioceptive discrepancy
							While both groups showed the effect of RHI in acceleration pattern in post-Illusion reaching trail, ASDs demonstrated a reverse pattern in acceleration of their movement, comparing to TDs
						Measure of the proprioceptive drift	Independent of condition, TDs showed more proprioceptive drift towards the rubber hand (or box) than the ASD group
						Measure of the skin temperature	Depending on the touch (synchronous vs asynchronous), TDs and ASDS showed different reaching profiles during RHI
						Reach trial task	No significant result for the relation of the skin temperature and the RHI
14	Kwakye et al. [174]	Altered auditory and multisensory temporal processing in ASDs	Expanding the previous finding of an extended temporal binding window for simple audio-visual input in ASDs by comparing the uni-sensory vs multisensory temporal processing within the sample of the ASDs children and adolescents	35 TDs children/adolescents (M:30, F:5; age Mean = 12.21 years, SD = 2.7)	27 TDs children/adolescents (M:22, F:5; age *M* = 11.73 years, SD = 2.4)	Visual temporal order judgment (TOJ)	No differences in thresholds for the visual TOJ task between ASDs and TDs
							Higher threshold on the auditory TOJ task in ASDs; suggestive of the altered auditory temporal processing. Specifically, ASDs required 48% more time between auditory stimuli to reliably determine which click occurred first
						Auditory TOJ task	ASDs showed a better performance within the Cross-modal (multisensory) ToJ task; suggestive of the extension of the temporal window for integration of the sensory information’s gathered across the different modalities
						Cross-modal version of TOJ task (to assess multisensory temporal function)	ASDs showed significantly more improvement in accuracy when compared to their TD peers for the 200 ms,300 ms, adding more support to the difference in size of the temporal binding window of the ASDs and TDs
							Regarding TDs, the improvements in response time happened over a greater range of delays relative to those for which TDs showed improvements in accuracy. However, ASDs still showed improvements in response time for approximately 100 ms longer than children with TD.
15	Foss-Feig et al. [175]	An extended multisensory temporal binding window in ASDs	Exploring the potential differences in multisensory temporal function in ASDs by inducing a low-level multisensory illusion with an audio-visual stimulus	21 ASDs children (*M* = 17, F = 4; age Mean = 12.60 years, SD = 2.6)	17 TDs children (*M* = 14, F = 3; age Mean = 12.09 years, SD = 2.2)	Flash-beep task (presentation of the single visual stimuli with simultaneous multiple auditory stimuli to induce the illusion of perception of multiple flashes)	ASDs showed a higher propensity to report the flash-beep illusion over an extended range of delays between the visual and auditory stimuli. This was evident by the approximate doubling in the range of the delays of the temporal binding window ASDs
							The lack of significant differences between ASDs and TDs for the control trials (e.g., one-flash/one-beep) was suggestive of a non-significant role of the response bias when it comes to accounting for the observed difference in temporal window size
							No significant differences between TDs and ASDs in reporting one flash in two-flashes/zero-beep conditions suggest that developmental differences in visual temporal acuity do not play a role in the highlighted perceptual differences between ASDs and TDs
16	Lafleur et al. [179]	Atypical implicit and explicit sense of agency in autism: A complete characterization using the cue integration approach	Obtain a comprehensive profile on the cognitive characteristics of the sense of agency (SoA) in ASDs	22 adults ASDs (*M* = 17, F = 5; age Mean = 30.14 years, SD = 6.53)	21 adult CA-matched TDs (*M* = 13, F = 8; age Mean = 27.71 years, SD = 6.01)	SoA psychophysics task in two conditions (operant vs observation)	Opposite to TDs, ASDs showed no significant differences in relative reproduction error (RRE- measure of the IB as an implicit sense of agency) across experimental conditions (operant and observation), suggestive of the absence of the temporal binding for action effect
			Explore the plausible demonstration of the alteration in ASDs in implicit and/or explicit levels of the cue-integration for the construction of Agency				Opposite to ASDs, TDs demonstrated a significant effect of social context on the binding strength
						Intentional binding measurement using a temporal reproduction method	In TDs, the Presence of an IB effect for pinching movements with visual outcomes for all operant conditions was indicated by the results.
			Investigate the possible alteration in the dynamic relation between the implicit and explicit sense of agency				In both TDS and ASDS, Feedback alteration had no impact on IB strength,
							In regard to the plausible correlation between implicit and explicit levels, ASDs showed a positive association between relative reproduction error (RRE) and explicit sense of agency (Judgment of agency)
17	Asada et al. [178]	Longitudinal study of personal space in autism	Comparing the size and the update of the interpersonal space over the course of the three years (during adolescence) between ASDs and TDs	13 ASDs (*M* = 12, F = 1; Time 1: Mean age = 15.4 years, SD = 1.6, range = 13.5–18.2; Time 2: Mean age = 18.4 years, SD = 1.6, range = 16.5–21.2)	9 TDs (*M* = 8, F = 1; Time 1: Mean age = 14.1 years, SD = 1.6, range = 12.7–16.4; Time 2: Mean age = 17.1 years, SD = 1.6, range = 15.7–19.4)	Interpersonal space task in two conditions (with or without eye contact)	Compared to TDs, the interpersonal space of ASDs was shorter both before and after the three-year interval.
							Opposite to TDs, ASDs showed a stable interpersonal space for the whole duration of the experiment (i.e., three years). This was indicative of the stability of the interpersonal space in ASDs, opposite to TDs, throughout adolescence.
18	Asada et al. [177]	Reduced Personal Space in Individuals with ASD	Comparing the personal space regarding humans and objects in TDs and ASDs	16 ASDs (*M* = 15, F = 1, age Mean = 15.7 years, SD = 1.6; range = 13.5–18.2 years)	16 TDs (*M* = 15, F = 1, age Mean = 14.4 years, SD = 1.9; range = 12.4–19.4 years)	Comfort-distance task (for measurement of the comfortable closeness to the experimenter or an object, involving 4 conditions (with or without eye contact + participant or experiment as an approaching agent).	Individuals with ASD showed significantly shorter interpersonal distances than TD individuals. This finding is suggestive of the presence of the altered regulation of the personal space regarding humans and objects in ASDs, as they showed ASD relatively smaller personal space in comparison to TDs.
							Regarding the effect of the eye contact, both TDs and ASDs showed significantly longer interpersonal distances when in the presence of eye contact.
			Exploring the plausible effect of the target (object vs humans), eye contact, and the score of the autistic trait on the size of the personal space.				A significant main effect of Target (object vs humans) was found since both TDs and ASDs demonstrated larger distances from the experimenter than from the object.
							No effect of age, IQ, autistic traits (SCQ and ADOS scores), and clinical complaints (CBCL scores) to the interpersonal distance results for both TDS and ASDs

*Abbreviations: ASD = Autism Spectrum Disorder; ASDs = individual diagnosed for autism TD/TDs = Typically Developing individuals; FBI = Full Body Illusion; AQ = Autism-Spectrum Quotient; QCAE = Questionnaire of Cognitive and Affective Empathy; QCAEcog = QCAE Cognitive Empathy subscale; NI = Numbness Illusion; MA = Mental Age; CA = Chronological Age; RHI = Rubber Hand Illusion; MIRAGE = name of the multisensory illusion apparatus used to induce spatial visuo-tactile conflict; SAS = Social Aptitude Scale; SZ = Schizophrenia; AS = Asperger Syndrome; HFA/HFAs = High-Functioning Autism/Autistic individuals; PDDNOS = Pervasive Developmental Disorder Not Otherwise Specified; SoA = Sense of Agency; IB = Intentional Binding; OP = Operant condition; CO = Control condition; PDS = Proportion Difference Score; MSI = Multisensory Integration

### Self-representation and bodily awareness in autism spectrum disorder

Studies of self-representation revealed a heterogeneous pattern in autism, with relatively preserved performance on some basic self-recognition and action-monitoring measures but differences in self-referential processing, self-other differentiation, and dynamic updating of self-related representations. Electrophysiological studies indicated altered neural processing of observed touch in ASD. Deschrijver et al. [181] found that high-functioning autistic adults displayed atypical event related potentials (ERPs) responses during action-based touch observation, with reduced animacy sensitivity and correlations between ERP amplitudes and sensory/social difficulties. Similarly, Noel et al. [182] demonstrated that peripersonal space boundaries in ASD failed to flexibly update in social contexts, contrasting with typically developed controls whose MSI adapted depending on social presence. Several studies addressed self-prioritization and ownership effects. Moseley et al. [183] reported that autistic adults exhibited enhanced accuracy for self-referential mismatches but attenuated friend-prioritization, with self-bias correlating positively with autistic traits. Reduced ownership effects in recognition memory tasks were observed by Grisdale et al. [184] and replicated in a preregistered study by Smith [185], both of which showed weaker memory advantages for self-owned items in ASD compared to typically developed individuals.

Findings on bodily and facial self-recognition also indicated differences. For example, Gessaroli et al. [129] reported that autistic children were faster at recognizing their own body parts compared to others but committed more errors overall. Deltort et al. [44] found that during a self-other morphing task, ASD adults demonstrated a lower threshold for recognizing self and altered responses to interpersonal multisensory stimulation, suggesting reduced susceptibility to the enfacement illusion. Using a novel double mirror paradigm, Lavenne-Collot et al. [186] further showed earlier self-recognition and delayed other-recognition in ASD adolescents, indicative of egocentric biases in self-other distinction. Delayed self-recognition was also a recurring theme. Dunphy-Lelii & Wellman [187] found that autistic children performed more poorly on delayed self-recognition and self-other action memory tasks compared to age-matched controls. Similarly, Dissanayake et al. [188] reported developmental delays in video-based self-recognition in children with high-functioning autism and Asperger’s disorder, although this ability improved with age. Carmody & Lewis [189] also observed reduced mirror self-recognition, personal pronoun use, and other-directed pretense in autistic children, with self-representation measures correlating with Autism Diagnostic Observation Schedule (ADOS) social interaction scores.

Studies on perspective-taking and own-body transformations suggested atypical reliance on non-embodied strategies in ASD. Conson et al. [190] showed that autistic adults relied more heavily on non-embodied cognitive strategies during visual perspective-taking, leading to underperformance in back-facing body conditions. Gauthier et al. [191] found that while own-body transformations were challenging across groups, children with ASD demonstrated less clear motor performance compared to both typically developed and developmentally coordinated peers. Likewise, Russo et al. [192] reported impairments in visual perspective-taking and body awareness in autistic children relative to both typically developed and intellectually disabled groups. Collectively, these findings; [44, 129, 181–192]; converge on the conclusion that ASD is associated with atypical self-referential memory, delayed and less flexible self-recognition, altered susceptibility to multisensory self–other illusions, and reduced embodiment in perspective-taking tasks. These atypicalities in self-processing may underlie difficulties in social cognition and interpersonal interaction commonly reported in ASD. In the following, summary of reviewed studies is presented in Table 2.

**Table 2:** Summary of empirical studies on self-representation in autism spectrum disorder (ASD), covering domains such as self–other distinction, self-referential processing, bodily self-consciousness, ownership effects in memory, self-recognition, and perspective-taking. The table outlines study aims, participant groups, experimental tasks, and key findings compared to typically developing (TD) and other control groups

Study summary			Aim of Study	Target group		Tasks	Results
NO	Authors	Title		Main group	Comparison Group/s		
1	Deschrijver et al. [181]	Action-based touch observation in adults with high functioning autism: Can compromised self-other distinction abilities link social and sensory everyday problems?	Investigate the relationship between impaired self-other distinction and social and sensory everyday challenges among ASDs by examining how ASDs process touch differently and how this affects their day-to-day activities	19 HFA adults (*M* = 13, F = 6, Mean age = 32.95 years, SD = 6.26)	17 CA-matched TDs (*M* = 11, F = 6, Mean age = 31.35 years, SD = 6.63)	Action-based congruency paradigm	TDs showed numerically larger P50 amplitude in wooden-hand congruent trials, compared to incongruent ones, while no congruency effect was shown in human-hand trials.
							HFAs showed a reverse numerical pattern in comparison to TDs in Wooden-hand conditions. Which means a smaller P50 amplitude in the congruent condition.
						EEG and ERPs recording	Although ASDs didn’t show any animacy difference between human-hand conditions and wooden-hand conditions, TDs showed larger N100 amplitude for human-hand trials as compared to wooden-hand trials
							The P3-index in the HFAs showed a negative correlation with sensation avoidance and a marginally significant negative relationship with sensation sensitivity. These findings suggest a relationship between reduced P3, in HFA, and observed sensory and social difficulties in the life of autistic individuals
2	Moseley et al. [183]	Levels of Self-representation and Their Socio cognitive Correlates in Late-Diagnosed Autistic Adults	Studying how the self-prioritization effect (SPE) and friend-prioritization effect (FPE) differ in individuals with autism and neurotypical individuals while they perform low- and high-level perceptual assignments	120 ASD adults (Mean age = 40.7 years, SD = 13.4)	44 TDs (Mean age = 26.7 years, SD = 8.6)	Matching task adapted from Sui et al. (2012) for online delivery via Tatool	ASDs showed significantly greater accuracy than the control group for Self-Mismatching items and Stranger-Matching items while there were no group differences for the critical Self-Matching items
							In contrast to TDs, who place themselves and a friend at the same distance from a stranger, autistic participants tend to perceive the distance between themselves and a stranger as significantly bigger than the distance between a friend and a stranger
							Higher levels of self-bias were linked to higher levels of autistic characteristics
			Examining connections between variations in self- and friend-representation and the social challenges connected with autism			Personal Distance Scale (PDS)	There were no differences in the accuracy of the ASDs between the Friend-Matching and Stranger-Matching tasks. However, they performed considerably better on the Self-Matching trial than the other two trials.
							Higher scores on Self-Matching items were accompanied by an advantage in Friend-Matching over Stranger-Matching tasks for the control group
3	Gessaroli et al. [193]	Self-face and self-body recognition in autism	Determine whether implicit self-body-parts and self-face-parts recognition can be observed in autistic children	17 ASDs children (chronological age: Mean = 12.8 years, SD = 3.7)	First group: 20 MA-matched TDs (TD1; chronological age: Mean = 8.5 years, SD = 2.21)	Visual matching-to-sample task	Regarding others’ facial and body parts recognition, children with ASD performed greater on tasks involving their own body and facial parts, indicating that they can discriminate between their own and others’ inputs and process self-stimuli more quickly.
							A higher error rate was observed in matching tasks for autistic children (12%) compared to TD1 (4%) and TD-2 children (3%)
					Second group: 18 CA-matched TDs (TD2; chronological age: Mean = 13.6 years, SD = 2.4)	The experiment consisted of two blocks (48 trials each), one with body-parts and the other block with face-parts	Sense of ownership was significant in both Body-Part-Type and Face-Part-Type. the variable Body-Part-Type was not significant whereas the variable Face-Part-Type was significant (eyes = % 14 vs. mouth = 8%)
							The two control groups were not statistically different and the variable Ownership) was significant in both of them
4	Grisdale et al. [184]	Self-referential memory in ASD and typical development: Exploring the ownership effect	Experiment 1: replication of the previously observed ownership effect in neurotypical adult	EXP1: no ASDs	EXP1: 40 TDs (*M* = 2, F = 38, age range = 18–24 years)	Recognition memory test	Although the mean corrected hit rate for self-owned items was higher than the mean corrected hit rate for other-owned items in neurotypical participants showing the expected ownership effect, this difference wasn’t observed in the ASD group indicating the disturbed ownership effect in autistic individuals
			Experiment 2: Investigate the association between ownership effect and sub-clinical levels of ASD characteristics and the degree of psychological self-awareness	EXP2: 16 ASDs adult (*M* = 13, F = 3, age Mean = 31.33 years, SD = 9.91)	EXP2: 16 TDs adults (*M* = 14, F = 2; age Mean = 35.31 years, SD = 14.70)	Private Self-Consciousness Scale (PSC)	The ASD and TD groups showed no differences in recognizing self-owned items, recognizing other-owned items, false alarm rates, and PSC scores
			Experiment 2: Explore the ownership effect in Memory and the diminishing of psychological self-awareness (measured using a questionnaire) in ASD individuals			Autism-spectrum Quotient (AQ)	No significant correlations between ownership (difference) score and PSC scores, while AQ scores and the size of the ownership effect were negatively correlated
5	Deltort et al. [44]	The enfacement illusion in ASD: How interpersonal multisensory stimulation influences facial recognition of the self	Evaluate the performance of self-recognition in ASD and Neurotypical adults during executing self-other discrimination task	15 ASDs, without intellectual disabilities (*M* = 15, F = 0; age 18–54 years, Mean = 28.5, SD = 11)	15 TDs adults (*M* = 15, F = 0; age 18–54 years, Mean = 28.5, SD = 10)	Face discrimination task by watching the morphing movie	Regarding stopping the morphing sequence, in the Self to-Other direction, the ASD group stopped when the face contained on average 45.9% of Self-face while the TD group stopped when the face contained on average 55.4% of Self-face
							In self-other recognition assessment, ASD group had a significantly lower mean Delta-POF (Percentage of Other Face content) than the TD group
							A significant main effect of the direction of morphing (Self to Other & Other to Self-direction) was observed in the ASD group. However, this effect wasn’t found in the TD group
			Investigating the effect of interpersonal multisensory stimulation (IMS) (i.e., enfacement illusion) on self-recognition performance in ASD and Neurotypical adults			IMS session	In the Self to-Other direction, a statistically significant difference in mean POF (Percentage of Other Face content) between TD and ASD groups was found because the mean POF was 9.456 points higher for the ASD group than for the TD group. Moreover, ASD participants had a significantly higher mean Delta-POF for the “Other to Self” direction, than in the “Self to Other “direction, by 5.1 points
							In contrast to the ASD group, the stimulation of the IMS (synchronous OR asynchronous) had a statistically significant effect on Delta-POF for the TD group
							IMS creates an enfacement illusion in individuals within the TD group only in the synchronous condition. By particularly affecting the recognition of the self-face only when the direction of morphing was a face transition from other to self
6	Dunphy-Lelii and Wellman [187],	Delayed self-recognition in autism: A unique difficulty?	Examining impaired self-recognition process and self-other action memory in autistic children	20 ASDs children (*M* = 18, F = 2; chronological age: *M* = 87.20, SD = 26.29 months; range = 5 years 1 month–13 years 7 months)	25 three-year-old children (*M* = 10, F = 15; chronological age: *M* = 41.80, SD = 1.80 months; range = 3 years 1 month–3 years 9 months)	Delayed self-recognition (DSR)	In the self-other action memory test, the combination of self and other scores (Actor sort score) in children with ASD had a positive correlation with DSR scores and also significantly lower than the two control groups
					27 five-year-old children (*M* = 19, F = 8; chronological age: *M* = 62.67, SD = 4.09 months; range = 4 years 8 months–5 years 10 months)	Self-other memory task	No correlation between the DSR score and the Bead Memory score was found in all the groups. Moreover, the Picture Similarity performance of autistic children wasn’t associated with their performance on the DSR task
						Standardized assessment of cognitive functioning via Bead Memory and Picture Similarities	No significant differences were observed between all the participants in the DSR scores
7	Gauthier et al.[191]	Behavioural Own-Body-Transformations in Children and Adolescents with Typical Development, ASD, and Developmental Coordination Disorder	Investigating the trajectory of behavioral OBT (own-body-transformation)	25 children and adolescents with ASD	36 TD children and adolescents	Own-Body-Transformation Guided Task (OBT Guided Task)	OBT was feasible but challenging for children in ASD and DCD groups; even neurotypical children experienced this phenomenon when the completion of the tasks depended on performing a mental rotation to take on a hetero-cantered perspective
							In terms of hetero-centered-perspective scores, the ASD and TD groups’ scores were highly similar and connected with age, TW orientation, and latency, however, the DCD group’s scores were significantly different from other groups due to impaired visual-spatial abilities.
			Development and the possible alteration in children with ASD and Developmental Coordination Disorder (DCD)	14 Children with Developmental Coordination Disorder (DCD)		The Tightrope Walker (TW) Paradigm	Children in the DCD group showed greater motor performance (measured by movement amplitude) than children with ASD due to more unclear and unreadable responses from individuals with ASD
							In the TW movements imitation was often not performed unambiguously or not at all. {19% (for TD), 25% (for DCD), and 39% (for ASD)}. Consequently, the ASD and TD groups demonstrated a high percentage of tagged movements (respectively 61 and 81%), which were only performed on succeeded imitation. On the other hand, the DCD group had an average position of 75% of tagged movements.
8	Russo et al. [192]	Exploring Visual Perspective Taking and body awareness in children with ASD	Studying Visual Perspective Taking (VPT) and Body Awareness performance in ASD and children with Intellectual Disability (ID) in comparison to neurotypical children (TD).	20 ASDs children (*M* = 14, F = 6; mean age = 5.5 years, SD = 2.04)	20 TD children (*M* = 11, F = 9; mean age = 4.6 years, SD = 1.05)	VPT task	Concerning body awareness, Children with ASD or ID showed more impairment compared to neurotypical children
						Body Awareness test	The mean VPT scores were significantly different and lower in the ASD group compared with the ID and control groups.
				20 children with Intellectual Disability (ID) (*M* = 13, F = 7, mean age = 5.1 years, SD = 1.92)			Self-pointing and self-naming scores were significantly higher in ASD and ID groups compared with the control group. Moreover, the performance on the VPT task was correlated with Self-pointing scores.
9	Noel et al. [182]	Inflexible Updating of the Self-Other Divide During a Social Context in Autism; Psychophysical, Electrophysiological, and Neural Network Modeling Evidence	Exploring the boundary between the self and others and its updating process in different social situations contexts in Autism	20 young adults with ASD (*M* = 12, F = 8; mean age = 21.3 years, SD = 4.5) 30 age- and gender-matched neurotypical young adults (*M* = 17, F = 13; mean age = 20.7 years, SD = 4.1)		High-density electroencephalography (EEG)	In ASDs group, in contrast to TDs, PPS does not shrink in the presence of others
			Investigating the social updating of peri-personal space from neurological aspects by using EEG			Visuo-tactile reaction time task (to measure PPS)	EEG recordings propose that only in the TD group, the alteration of the multisensory integration process depended on the social context
			Proposing an account of the underlying neural circuitry that may be distinct in ASD vs. neurotypical controls				Visuo-tactile disparity had a strong effect on the multisensory responses, the less distance between the presentation of touch and vision led to strong multisensory integration.
10	Conson et al. [190]	“Put Myself Into Your Place”: Embodied Simulation and Perspective Taking in ASDs	Exploring the plausible alteration of ASDs in perspective taking ability and investigating the neural and cognitive underlying reasons within the context of embodied simulation theory	22 high-functioning ASD adolescents (*M* = 20, F = 2; age 12–16 years, *M* = 13.3, SD = 1.4 years)	22 age- and gender-matched TD adolescents (*M* = 20, F = 2; age 12–16 years, Mean = 13.5, SD = 1.5 years)	Visual perspective taking task	Opposite to TDs, ASDs rely heavily on the non-embodied strategies, which could be the potential underlying reason for the underperformance in the prospective-taking task, independent of direction bodies.
							Opposite to the back-facing bodies condition, ASDs outperformed TDs in front-facing conditions
						Own body transformation task with two directions (front-facing body vs back-facing body)	ASDs showed difficulties in cognitive control over the self- and other-perspective.
11	Carmody & Lewis [189],	Self-Representation in Children with and Without ASDs	Exploring the plausible alteration and the developmental delay of the self-representation abilities in ASDs children by comparing them to TDs infants	20 ASDs children (*M* = 17, F = 3, aged between 54 and 67 months, Mean = 57.85, SD = 3.57 months)	66 TDs infants (*M* = 33, F = 33, assessed within 1–2 weeks of 24 months of age)	Mirror Recognition	Overall, TDs showed a more advanced level of self-representation capability compared to ASDs, evident by the data that all TDs performed better on the combination of the mirror recognition task and behavioral tasks.
							Self-representation ability in children with ASD was correlated to their scores on the social Interaction subscale of the Autism Diagnostic Observation Schedule (ADOS), suggesting that underdeveloped Self-representation abilities in some ASDs could be an underlying reason for poor social interaction skills observed in this population.
						Other-Directed Pretend Play	TDs were more likely to show other-directed pretense (80%) than the ASDs (35%). Also, Personal pronouns were used more by TDs (83%) than by ASDs (63%). Also, TDs showed higher passing rate of the mirror recognition task compared to ASDs (100% vs 55%)
12	Lavenne-Collot et al. [186]	Self/other distinction in ASD adolescents assessed with a double mirror paradigm	Determine whether implicit self-body-parts and self-face-parts recognition can be observed in autistic children	7 ASDs Adolescence (*M* = 7, F = 0; age Mean = 13.8 years, SD = 2.17; range = 11.5–16.8 years)	7 age- and sex-matched TD adolescents (*M* = 7, F = 0; age Mean = 13.6 years, SD = 1.81; range = 11.5–16 years)	Morphing live-video task by the employment of the new double mirror paradigm based on the Alter Ego System, which consists of a semi-transparent double mirror in two directions (self-to-other vs other-to-self)	ASDs showed earlier self-recognition in the other-to-self sequence and delayed other-recognition in the self-to-other sequence, indicative of the presence of an egocentric bias in this population.
							The correlation between ASDs’ symptom severity and the impairment in the self/other distinction was marginally significant, evidenced by the earlier face recognition in more severely affected individuals.
							In the ASDs group, opposite to TDs, the main effect of the direction of the morphing on the critical threshold for switching between self and other was significant.
							In contrast to previous research that relied on static images, this study, by the use of a double mirror apparatus for morphing, provides new evidence supporting the role of bodily self-consciousness in understanding the self/other distinction impairment
13	Smith et al. [185]	Revisiting the ownership effect in adults with and without autism	Exp1: Replication of previous findings of the presence of the ownership effect and its correlation to the self-reported autistic traits (AQ) by using a pre-registered design and analysis	Exp 1: no ASDs	Exp 1: 100 TDs (*M* = 16, F = 84, age 18–58 years, Mean = 20.11, SD = 4.80 years)	Memory-ownership task (presenting 222 pictures of items which are divided into 3 groups: “self-owned items”, “other-owned items”, and “lure items” to participants while each item is colored differently depending on the group they belong to). Participants have to sort these items into their groups in the recognition phase while the items are presented without any color.	ASDs showed a significantly reduced ownership effect compared to age- and IQ-matched TDs, evident by the statistically insignificant ownership effect in this population
			Exp 2: Investigating the possible alteration in self-referential cognition of ASDs in comparison with TDs, by employing a memory-specific task for assessing the differences in the size of the ownership effect as one of the indicators of the self-reference effect.	Exp 2: 28 ASDs (*M* = 19, F = 9; Mean age = 38.77 years, SD = 12.69)	Exp 2: 28 age- gender-and IQ-matched neurotypical adults (*M* = 18, F = 10; Mean age = 39.47 years, SD = 14.18)		While there was no significant group difference in memory for other-owned items, TDs had higher memory for self-owned, compared to ASDs.
							TDs showed better memory for self-referent information (self-owned items) than information relating to others. This is indicative of the unaltered presence of self-bias, or “self-reference effect” in TDs.
14	Dissanayake et al. [188]	Delayed video self-recognition in children with HFA & Asperger	Comparing the performance of the ASDs, TDs, and children with Asperger’s disorder within the delayed video self-recognition (DSR)	Exp1: 15 HFAs children (*M* = 15, F = 0; age range: 5–9 years, Mean = 7.58, SD = 0.98 years)+ 12 children with Asperger’s disorder (*M* = 12, F = 0; age range: 5–9 years, Mean = 7.31, SD = 1.15 years)	Exp1:15 CA-matched and MA-matched TD Children (*M* = 15, F = 0; age range: 5–9, Mean = 6.99, SD = 1.20 years)	Control Video task	In Exp1, all ASDs were able to understand the correspondence between the delayed video image and current reality
						Delayed self-recognition task (DSR)	In both experiments, the groups were not different on the DSR task
			Exploring the possible dependency of the DSR ability on metarepresentational capacity	Exp2: 18 HFA children (*M* = 15, F = 3; age range: 4–7 Mean = 5.65, SD = 1.13 years)	EXP2: 18 TDs children (*M* = 14, F = 4; *M* = 14, F = 4; age range: 4–7, Mean = 4.99, SD = 0.81 years)		Compared to Exp1, in Exp2, fewer HFAs responded to their unmarked video image with a pronoun compared to the TDs, who all responded appropriately. Since the HFAs sample was younger in the second experiment, these results show the trajectory of the developing DSR capability in HFAs children according to age
						Theory of minds task	In all groups, none of the results supported the hypothesized association between DSR performance and meta-representational ability
							In all groups, no supportive evidence was seen for any correlation between personal pronoun use and DSR score

* Abbreviations: EEG = Electroencephalography; ERP/ERPs = Event-Related Potential(s); SPE = Self-Prioritization Effect; FPE = Friend-Prioritization Effect; PDS = Personal Distance Scale; PSC = Private Self-Consciousness Scale; OBT = Own-Body-Transformation; DCD = Developmental Coordination Disorder; TW = Tightrope Walker paradigm; VPT = Visual Perspective Taking; ID = Intellectual Disability; DSR = Delayed Self-Recognition

** Abbreviations introduced under Tables 1 are not repeated here; readers are referred to the abbreviation lists under Tables 1 for unfamiliar terms

### Motor function, action organization, and body awareness in autism spectrum disorder

Across the five studies, motor findings were heterogeneous but indicated recurring differences in prospective action organization, contextual modulation of movement, and body awareness in autism. Chua et al. [194] reported longer movement times, lower peak velocity, and greater reliance on corrective movement units in autistic children, with group differences varying with age and target distance. Dowd et al. [195] found greater variability in movement preparation but relatively preserved execution, together with reduced modulation of movement by a visual distractor. Minissi et al. [196] found task-dependent whole-body motor differences, which emerged during the precise flower-picking task but not during kicking or bubble tasks. Palmer et al. [197] found broadly comparable perceptual rubber hand illusion effects across groups but reduced illusion-dependent modulation of subsequent reaching movements in ASD and high-AQ participants. Finally, Bertilsson et al. [198] identified difficulties in body awareness, coordination, movement quality, and bodily control in autistic young adults. Together, these findings identify differences in motor organization and bodily awareness but do not establish motor measures as autism-specific diagnostic biomarkers. In the following, a summary of the reviewed studies is presented in Table 3.

**Table 3:** Summary of empirical studies on motor function and action organization in autism spectrum disorder (ASD), focusing on goal-directed movement, kinematic strategies, context sensitivity, and body awareness. The table outlines study aims, participant groups, experimental tasks, and main findings concerning motor and bodily processes in autistic individuals, including comparisons with TD or other comparison groups where applicable

Study Summary			Aim of Study	Target groups		Tasks	Results
NO	Authors	Title		Main group	Comparison group/s		
1	Chua et al. [194]	Developmental differences in the prospective organization of goal-directed movement between children with autism and typically developing children: A smart tablet serious game study	Exploring the kinematic strategies used by ASDs and TDs and investigating whether these possible differences could be used as potential biomarkers for ASD, through the use of a smart tablet serious game for executing goal-directed movement	28 autistic children (Mean = 17, F = 11; age *M* = 4.7 years, SD = 0.9; range = 2.8–6.6 years)	43 TD children (*M* = 30, F = 13; age Mean = 4.7 years, SD = 0.9; range = 3.0–6.2 years)	A smart tablet serious game called the “Sharing” game to assess goal-directed movement	ASD children showed longer movement time and time-to-peak velocity, lower peak velocity, and more corrective movements than TD children. Group differences also varied with age and target distance, suggesting greater reliance on feedback control.
2	Minissi et al. [196]	The whole-body motor skills of children with ASD taking goal directed actions in virtual reality	Exploring the differences in the whole-body motor skills between ASDs children and TDs during the execution of three tasks similar to everyday activities within in virtual reality	20 ASDs (*M* = 16, F = 4; age Mean = 53.44 months, SD = 13.22)	20 TDs (*M* = 10, F = 10; age Mean = 59.40 months, SD = 11.95, age range = 3–6 years)	Three different tasks in virtual reality	Group differences were task-specific. ASD children showed greater whole-body and upper-limb movement abnormalities and longer completion time during the precise flower-picking task, but not during the kicking or bubble tasks.
						(Ball-kicking task, Flower-picking task, Bubble-blowing task)	
3	Dowd et al. [195]	Do Planning and Visual Integration Difficulties Underpin Motor Dysfunction in Autism? A Kinematic Study of Young Children with Autism	Exploring the possible alteration in the upper-limb motor function of young children with autism by measuring the point-to-point upper-limb movement kinematics in ASDs and TDs.	11 high-functioning ASDs (*M* = 8, F = 3; age Mean = 6.2 years, SD = 1.4)	12 CA-matched TDs (*M* = 9, F = 3; age Mean = 6.6 years, SD = 1.5)	Point-to-point movement paradigm by using a tablet kinematic task with adaptation from the distractor inference task	ASD children showed greater variability in movement preparation but relatively intact movement execution. Unlike TD children, their movement planning and execution were minimally affected by a visual distractor.
4	Palmer et al. [197]	Context sensitivity in action decreases along the autism spectrum: a predictive processing perspective	Investigating the plausible difference in the sensitivity to the preceding sensory information in motor function in ASDs, compared to TDs (with high and low autism-spectrum quotient)	15 ASD (*M* = 11, F = 4; age Mean = 29.27 years, SD = 9.17)	15 TDs with low autism-spectrum quotient (AQ, *M* = 7, F = 8; age Mean = 30.20 years, SD = 7.31)	RHI	All groups showed the expected RHI, with stronger illusion following synchronous than asynchronous stimulation; perceptual susceptibility was bodily similar across groups.
						Reach to grasp movement (for kinematic recording)	Low-AQ participants showed RHI-related changes in movement kinematics, whereas high-AQ and ASD groups showed little kinematic modulation by the illusion, indicating reduced sensitivity to prior bodily context. The ASD group also showed greater overall movement jerk and delayed early movement features.
						Measure of the perceptual feeling of illusion + illusion onset latency + Proprioceptive drift	Illusion ratings were higher after synchronous than asynchronous stimulation in all groups, with the same general pattern across low-AQ, high-AQ, and ASD participants.
					15 TDs with high autism-spectrum quotient (AQ, *M* = 10 F = 5; age Mean = 29.87 years, SD = 8.61)		
							No significant group difference in the time required to experience the illusion during synchronous stimulation.
							Synchronous stimulation produced greater drift toward the rubber hand than asynchronous stimulation, with no significant group differences.
5	Bertilsson et al. [198]	Understanding one’s body and movements from the perspective of young adults with autism: A mixed-methods study	Exploring the ASDs’ experience regarding their body and movement, and the potential benefits of physiotherapy for this population	11 ASDs (*M* = 2, F = 9, median age = 19 years; range = 16–22 years)	None	A semi-structured face-to-face interview (for obtaining the subjective understanding of autistic individuals regarding their body and movement) while employing Bears Cards and five pairs of pictures describing body postures and movements to meet the needs of the participants	Difficulties were reported in body awareness, body control, coordination, and understanding bodily signals.
						Motor development was assessed using Bruininks–Oseretsky Test of Motor Proficiency	Motor proficiency was generally reduced, with better fine than gross motor functioning and reduced muscle strength.
						The quality of the movement and the subjective experiences were examined using the Body Awareness Scale Movement Quality and Experience (BAS MQ-E)	Difficulties in movement quality, body awareness, coordination, and postural control were identified.

* Abbreviations: BAS MQ-E = Body Awareness Scale – Movement Quality and Experience

** Abbreviations introduced under Tables 1 and 2 are not repeated here; readers are referred to the abbreviation lists under Tables 1 and 2 for unfamiliar terms

## Discussion

Across the 37 included studies, the most coherent pattern was not a global impairment of multisensory or self-related processing, but context-dependent differences in how sensory and sensorimotor information is weighted and used to update bodily self-representations. Differences were particularly apparent when tasks required multisensory temporal integration, flexible updating of body or interpersonal boundaries, incorporation of prior bodily context into action, or prospective motor control. At the same time, relatively preserved performance under some explicit, unimodal, or low-noise conditions argues against a unitary deficit account. We therefore interpret the literature in terms of differences in the flexibility and weighting of self-related sensorimotor information, while recognizing that developmental and methodological heterogeneity currently limits strong mechanistic and clinical conclusions.

### Altered multisensory integration in autism

While the importance of sensory processing in understanding autism is widely recognized [199–203], debates persist regarding the extent to which sensory alterations in autistic individuals affect local and global models of sensory processing. Local processing mechanisms are activated when detailed features must be analysed or intricate patterns discerned, and numerous studies have shown that autistic individuals often exhibit heightened sensitivity in these areas [201, 203, 204]. In contrast, global processing is essential for integrating inputs into coherent wholes, i.e., recognizing movement trajectories or spatial layouts. A review study [205], cautiously concludes that individuals with ASD show a marked preference for local over global processing, suggesting that sensory difficulties in autism may be especially pronounced in tasks requiring integration of contextual or holistic information (see also [206]).

Local-global processing accounts and multisensory integration accounts address related but distinct aspects of perceptual organization. The reviewed evidence suggests that autism-related differences may become particularly apparent when information must be integrated across sensory modalities or contextual sources [207]. In other words, the sensory alterations commonly associated with ASD may largely stem from deficits in MSI, which requires the synthesis of inputs from multiple sensory modalities. More specifically, as suggested by Masterton and Biederman [208], the imbalances in proprioceptive integration could be a core dysfunction in the sensory integration system of individuals with ASD.

In recent decades, efforts to better understand the mechanisms underlying autistic traits, particularly difficulties in social interaction, have increasingly focused on MSI and its potential influence on higher-order cognitive and social functioning. A range of experimental paradigms has been developed to examine MSI and its relationship with different aspects of self-representation (e.g. [84]). Among these, illusion-based paradigms, such as the rubber hand illusion, numbness illusion, and full-body illusion, are widely used to investigate multisensory processing and body ownership. Although studies have not consistently reported reduced susceptibility to perceptual illusions in autistic individuals [206, 209–213], these paradigms remain valuable for examining differences in MSI and their potential relationship with atypical embodiment in ASD.

Earlier dimensional studies suggest that variability in autistic traits and inadequate matching of comparison groups may contribute to inconsistent findings on illusion susceptibility, highlighting the importance of accounting for heterogeneity when interpreting group differences [206].

The rubber hand illusion has received particularly sustained attention as a paradigm for examining multisensory contributions to body ownership and the flexibility of bodily representation [65, 83]. Variations manipulating temporal, spatial, and anatomical congruence have further demonstrated that embodiment depends on the integration and weighting of visual, tactile, and proprioceptive information [214–222].

Variations of the RHI further suggest that embodiment differences in autism cannot be explained by a general absence of illusion susceptibility. Rather, effects appear to depend on the relative weighting of visual, tactile, and proprioceptive information and on the contextual demands of the paradigm [84].

Another particularly innovative variation of the rubber hand illusion involved the use of video goggles to reduce proprioceptive discrepancies and enhance immersion in the illusion [223]. Interestingly, while this technology increased the strength of the illusion in typically developed individuals, it had no significant effect on participants with ASD [84]. When these findings are considered alongside the results from standard rubber hand illusion paradigms, they suggest that individuals with high-functioning autism (HFA) may be less sensitive to visuo-tactile discrepancies. This supports the notion put forth by Happé and Frith [205] that while local and visuo-tactile integration in ASD may be preserved, difficulties emerge when more global, context-dependent integration is required, such as in the rubber hand illusion, which involves aligning visual and tactile inputs within a proprioceptive framework (see also [204]).

The aforementioned reduced sensitivity to visuo-tactile discrepancies is not necessarily permanent. While individuals with ASD typically demonstrate lower initial susceptibility to the rubber hand illusion, research shows that their illusion strength approaches that of typically developed individuals after prolonged exposure (approximately six minutes [170]). This delayed response has been attributed to an extended temporal window for MSI [174, 175, 224–226]. However, Noel et al. [128, 176] proposed an alternative explanation: individuals with ASD may preferentially integrate tactile and proprioceptive information over exteroceptive cues like visual or auditory stimuli. This preference aligns with earlier findings that individuals with ASD often rely more heavily on proprioceptive input and down-weight visual cues in visual-proprioceptive integration tasks [28]. Despite the large body of research supporting the alteration in MSI between ASDs, some studies have challenged this perspective by critiquing methodological limitations. For instance, Zaidel et al. [172] proposed that global processing and the MSI processes are intact in autistic individuals. They contended that reported deficits such as reduced audiovisual facilitation, unoptimized neural integration, and altered temporal processing [76, 227–229] are hardly suggestive of the true alteration of MSI in autistic individuals. Instead, these alterations may stem from co-occurring low-level auditory deficits [174, 230]. Additionally, they noted that many MSI studies have focused primarily on autistic children, whereas studies involving autistic adults often yield inconsistent findings (see [231–233]). The last critique raises concerns, as MSI is not fully developed until adolescence [228, 234], complicating interpretation across age groups [228, 234]. To add another explanation for the observed alteration of MSI in ASDs, Zaidel et al. [172] suggested that individuals with ASD may exhibit heightened sensitivity to sensory noise due to an over-reliance on incoming sensory input relative to prior expectations or top-down modulation [235, 236]. Regardless of the specific mechanism, the consistently observed lower susceptibility to the rubber hand illusion in ASD points toward difficulties in disembodiment, i.e., the ability to detach from one’s bodily self and adopt external sensory cues. This is supported by evidence of a steeper self-to-other representational gradient in ASD [128, 168], which may also explain the sharper boundaries of peripersonal space, diminished perspective-taking abilities, and reduced responsiveness to bodily illusions compared to neurotypical individuals.

Beyond its role in investigating altered body representations, the rubber hand illusion has also been used to test broader hypotheses concerning autism-related differences in social and perceptual processing. One such hypothesis, proposed by Kaiser and Pelphrey [237], is that individuals with ASD may be less sensitive to distinctions between biological and non-biological motion, a possibility supported by earlier findings (e.g. [238]). This hypothesis has been examined indirectly using the rubber hand illusion by manipulating the source of tactile stimulation. In Paton et al. [84], touch was delivered either by a human experimenter or by a mechanical device. Although both typically developing and autistic participants experienced a weaker illusion during mechanically delivered touch, no significant group difference was observed. These findings therefore do not support a specific reduction in sensitivity to biological motion cues in autistic individuals within this context.

A further challenge in interpreting rubber hand illusion findings concerns the extent to which different outcome measures reflect the same underlying aspect of embodiment. Subjective questionnaires capture the conscious experience of ownership, whereas proprioceptive drift provides a behavioral index of changes in perceived limb location [218, 239]. Importantly, these measures should not be treated as interchangeable. Proprioceptive drift does not always correlate with subjective illusion strength [90], and drift can occur in the absence of a corresponding subjective illusion [240–242]. At the same time, a recent meta-analysis reported a significant association between proprioceptive drift and illusory embodiment [243]. Together, these findings indicate that proprioceptive drift remains informative but is better interpreted as a complementary behavioral measure of multisensory recalibration rather than as a direct or exclusive measure of body ownership. The sensitivity of drift to experimental procedures is also important, as differences between synchronous and asynchronous conditions may depend on the temporal characteristics of the asynchronous stimulation [84].

This distinction is particularly informative in autism. Earlier proprioceptive studies in typically developing individuals demonstrated systematic biases in limb localization when visual information was unavailable [244–247]. In comparison, Paton et al. [84] found that autistic participants localized their real hand more accurately than typically developing participants when vision was occluded, independently of subjective illusion strength. This pattern is compatible with greater weighting of proprioceptive information in autism. Such findings can be considered alongside weak central coherence accounts, which emphasize preferential processing of local relative to broader contextual information [201, 204, 205, 248], and enhanced perceptual functioning accounts, which emphasize increased processing of local perceptual information [203, 249]. Greater precision of proprioceptive localization may consequently contribute to differences in sensorimotor control and movement planning [84, 250–252].

Other proposed implicit or objective measures show similar limitations. Changes in movement parameters following rubber hand illusion induction have been inconsistent in neurotypical participants [242, 253–255], although movement-related differences have been reported in autism, including altered acceleration following synchronous relative to asynchronous stimulation [20, 84, 121–123]. These findings are broadly compatible with accounts proposing altered weighting of sensory evidence and prior expectations during movement control [203, 205, 235, 250–252]. Skin temperature has also been proposed as a physiological index of embodiment [256], but its interpretation remains uncertain because cooling of the real hand has not been consistently replicated [84], and cooling may itself facilitate illusion onset rather than result from the illusion [257]. Taken together, these findings support the use of multimethod assessment, combining subjective ownership, proprioceptive localization, movement kinematics, and physiological measures, rather than treating any single measure as a definitive index of embodiment.

The preceding evidence illustrates the variety of rubber hand illusion paradigms and outcome measures used to investigate body ownership in autism, revealing both similarities and differences relative to non-autistic populations. Alongside the rubber hand illusion, researchers have also employed the numbness illusion as a complementary paradigm for examining multisensory integration and bodily self-processing. An important distinction between these paradigms concerns the relative contribution of different sensory modalities. Whereas the rubber hand illusion relies primarily on visuotactile integration within a proprioceptive spatial framework [84], the numbness illusion depends more directly on the integration of tactile and proprioceptive information. The numbness illusion paradigm also allows manipulation of two important factors: whether touch is self-generated or other-generated and whether the tactile stimulation is synchronous or asynchronous. These manipulations have enabled researchers to examine the effects of self- and other-generated touch in both autistic and non-autistic participants [75, 258, 259]. Two notable findings have emerged from this literature. First, autistic participants have been reported to experience the numbness illusion during both synchronous and asynchronous stimulation when the touch is self-generated. Second, the strength of the illusion during other-generated touch appears broadly comparable between autistic and typically developing groups and is generally weaker than during self-generated touch, irrespective of synchrony. Taken together, these findings suggest that differences in multisensory integration in autism are not uniform across sensory combinations or task conditions. In particular, some aspects of tactile modulation associated with self-generated action appear relatively preserved [260], emphasizing the importance of distinguishing the sensory and agency-related demands of different embodiment paradigms.

#### Multisensory integration: limitations and future directions

A central methodological limitation is that embodiment paradigms such as the rubber hand illusion potentially affect several related but distinguishable bodily representations. Visuotactile conflict can influence body ownership, body schema, body image, and action-related spatial representations [261–267]. Because imitation and action also depend on dynamically maintained representations of the self and others [199], differences observed during RHI paradigms cannot automatically be attributed to a single construct such as ownership. This is particularly important in autism, where body schema, sensorimotor integration, and action organization may also differ [84, 121, 268]. Although the RHI has consequently been described as involving body-schema-related recalibration [269], future studies should distinguish more explicitly which component of bodily representation each dependent measure is intended to assess.

Future research should examine whether differences in MSI are associated with reduced flexibility in the updating of bodily self-representations. Experimental manipulations may also help establish whether these representations are modifiable. For example, multisensory training has been proposed as a means of altering the temporal or spatial weighting of sensory information and potentially the flexibility of peripersonal and self-other boundaries [128]. Tool-use paradigms provide another way of experimentally modifying action-related body and spatial representations, although effects remain heterogeneous even in neurotypical populations [270–273]. These approaches should currently be regarded as experimental tests of plasticity rather than established interventions.

A further limitation is the absence of a single theoretical account that explains the variability across MSI and embodiment paradigms. Findings may reflect enhanced processing of local sensory information [203], preferential weighting of local over global contextual cues [205], or different contributions of these mechanisms across individuals and tasks. Methodological factors, including small and heterogeneous samples, may further contribute to inconsistent results [206]. Larger samples, continuous-trait approaches, and paradigms that manipulate sensory reliability directly will be important for distinguishing these explanations.

### Self-bias and self-other differentiation in autism

The inquiry about the nature of the altered multimodal processes within the autistic community is not confined to the study of underlying processes of body representation modulations through rubber hand illusion, numbness illusion, and full body illusion studies. Another domain thought to reflect atypical multimodal sensory integration in ASD is self-face recognition. For instance, Platek et al. [274] found that in typically developing adults, prior exposure to other sensory modalities such as hearing one’s name or smelling one’s scent facilitated faster recognition of one’s own face, but not of familiar faces. This finding suggests that self-face recognition relies on a multimodal representation of the self, unlike recognition of familiar others, which is primarily visual.

Early studies suggested that basic mirror self-recognition can emerge in autistic children at a mental age of approximately two years [275, 276], despite differences in cue use, configural processing, and memory for faces [277–279]. However, interpretation of these studies is constrained by small samples, inadequate mental-age matching, and variation in cognitive ability [276, 280, 281]. Moreover, successful mirror self-recognition does not necessarily establish the presence of a fully developed or temporally extended self-representation [187, 280, 282]. More recent paradigms therefore combine mirror recognition with delayed self-recognition, action-memory, and self-other morphing tasks to distinguish basic recognition from self-other differentiation and dynamic updating of self-representation [44, 187, 283, 284]. Collectively, these findings suggest that relatively preserved basic self-recognition can coexist with differences in temporally extended or dynamically updated self-representation.

Delayed self-recognition should also be interpreted cautiously because performance depends on cognitive processes beyond self-representation. Although successful delayed recognition has been interpreted as evidence for a temporally extended self-concept [188, 284, 285], autistic children may use alternative strategies to solve these tasks [286, 287]. Conversely, failure may partly reflect differences in episodic or autonoetic memory rather than self-representation itself [288, 289], consistent with associations between delayed-self-recognition performance, recall, and verbal abilities [290–292]. Developmental stage is therefore critical because autonoetic memory and delayed self-recognition continue to develop substantially between three and five years even in typically developing children [293–295]. Studies using both chronological-age and mental-age comparison groups may therefore be particularly informative for distinguishing developmental delay from autism-specific differences [187, 188, 284].

Neuroimaging findings provide a complementary level of evidence. Although broadly similar self-processing regions are recruited in autistic and non-autistic participants, differences in activation magnitude and recruitment have been reported [296], including increased right prefrontal and inferior frontal activity during some self-referential tasks [297, 298]. These findings suggest that broadly overlapping neural systems may support self-processing across groups while differing in their functional recruitment or the strategies used to perform the task.

Deltort et al. [44] help bridge the apparent gap between relatively preserved behavioral self-face recognition and differences observed at neural or multisensory levels. Using bidirectional self-other morphing combined with interpersonal multisensory stimulation, they found direction-dependent differences in self-recognition in autistic participants and reduced modulation of self-identification by synchronous visuotactile stimulation. This paradigm extends earlier work on the enfacement illusion [299, 300] and suggests that basic recognition of one’s own face may remain relatively preserved while the multisensory updating of self-face representations differs. This interpretation is broadly compatible with reduced or delayed modulation reported in other bodily-illusion paradigms, including the RHI [84, 170], full-body illusion [168], and numbness illusion [75], although the mechanisms underlying these paradigms should not be assumed to be identical.

#### Theoretical accounts of altered self-face recognition and self-other discrimination in autism

The heterogeneous findings in self-face recognition may be interpreted in relation to at least two partially overlapping perceptual mechanisms. Enhanced Perceptual Functioning accounts emphasize increased reliance on local perceptual information in autism [203], while developmental studies indicate differences in attention allocation and face-specific processing [277, 301–303]. A persistent analytical processing strategy may also reduce reliance on the configural processing commonly used for familiar and self-faces [304–306]. Differences in the enfacement illusion may additionally reflect variation in multisensory integration rather than face processing alone.

These perceptual differences may also influence how self-representations are updated over time. Dynamic self-face representation depends on plasticity within multimodal regions involved in self-face processing and first-person bodily representation [307, 308]. Interpersonal multisensory stimulation can modify self-face representations in neurotypical participants, as demonstrated by the enfacement illusion [44]. Differences in this form of updating in autism may therefore reflect variation in how multisensory information is incorporated into an existing self-representation. Related differences have been reported in the enfacement illusion [44], RHI [84, 170], full-body illusion [168], and numbness illusion [75]. Together, these findings motivate the hypothesis that the flexibility of multisensory updating, rather than basic self-recognition itself, may be particularly informative in autism.

Dynamic self-representation may also intersect with broader socio-emotional processes. Differences in facial expression processing, resonance, and emotion recognition have been reported in autism [309–311], together with differences in self-emotion identification and online social cognition [31, 312]. Associations between susceptibility to bodily illusions, empathy, and autistic traits have also been reported [168, 170]. These relationships are consistent with interaction between bodily self-processing and socio-emotional cognition, but they do not establish that altered multisensory self-updating causes social-cognitive differences.

Self-related processing has been studied using several related but conceptually distinct effects. Self-prioritization or self-advantage refers broadly to preferential processing of self-related stimuli [313–315], supported by evidence that self-generated and other-generated information can recruit different neural processing pathways [316–319]. Memory-based paradigms examine related but distinct processes. Self-other action-memory tasks assess memory for self-generated versus other-generated actions and therefore involve self-other differentiation and agency-related processing [187], whereas ownership-memory paradigms examine whether assigning an item to the self, enhances subsequent memory [185]. Visual matching and hand-related tasks provide still other indices of preferential self-processing and its relationship with body representation [193, 320, 321]. Distinguishing these paradigms is important because preserved performance on one form of self-advantage does not necessarily imply preserved self-representation across all domains.

#### Self-bias and self-other differentiation: limitations and future directions

Interpretation of self-bias and self-other differentiation is constrained by substantial variability across tasks and age groups. Some studies report a reduced or absent self-reference advantage in autistic children, whereas others find comparable self-other action memory, online monitoring, or visual matching performance [31, 193, 322]. Similarly, findings in adults range from diminished self-reference effects to preserved or enhanced memory for self-experienced relative to observed events [323–325]. One likely source of heterogeneity is that implicit and explicit tasks capture different aspects of self-processing [326]. For example, self-advantage effects may emerge in implicit sensorimotor tasks even when explicit self-other discrimination does not differ [321], consistent with the possibility that ongoing sensorimotor updating contributes differently across paradigms [190, 193].

An additional framework for integrating these findings is interpersonal synchrony, broadly defined as the temporal coordination of behavior or activity between interacting individuals [327, 328]. Interpersonal synchrony can occur at multiple levels, including behavioral or motor, conversational, physiological, and neural synchrony [328]. Within the behavioral and motor domain, synchrony may arise spontaneously during interaction or intentionally when individuals deliberately coordinate their actions, and it may involve rhythmic movements as well as less structured coordination of gaze, gesture, posture, or facial behavior [327]. Interpersonal synchrony should also be distinguished from simple imitation, because synchrony concerns the temporal coupling of interacting partners and does not necessarily require the production of identical actions. Studies in autism have reported differences in both spontaneous and intentional motor synchrony, as well as reduced nonverbal movement synchrony during naturalistic social interaction, although findings vary across paradigms and participant groups [329–332]. In the context of the present review, behavioral and motor synchrony are particularly relevant because temporal coordination between one’s own actions and those of another person requires ongoing integration of sensory information about self-generated and other-generated events. Interpersonal synchrony may therefore provide a bridge between multisensory integration, agency, and higher-order self-other differentiation [327]. From this perspective, differences in sensitivity to temporal or spatial correspondence could influence the dynamic calibration of self-other boundaries during interaction. Future studies should examine these relationships within genuinely interactive paradigms in which different forms of interpersonal synchrony are experimentally manipulated rather than inferred from isolated multisensory tasks.

The enfacement and rubber hand illusions both depend on temporally coordinated multisensory signals, but differences in susceptibility should not be interpreted as evidence for a unitary impairment in cross-modal integration. Deltort et al. [44], for example, suggest that the dynamic updating of self-face representation may differ even when basic self-recognition remains relatively preserved. Future studies should therefore combine multisensory self-face paradigms with established assessments of configural face processing, including the inverted-face effect [333], composite-face procedures, and morph-based recognition tasks [334–336]. Such designs may help distinguish general face-processing differences from mechanisms more specifically related to multisensory self-representation. While these explanations are supported by empirical findings [44], further research is necessary to deepen our understanding of self-face recognition in autism. In particular, future studies should include larger sample sizes and incorporate well-established paradigms used to assess configural face processing, such as the inverted face effect [333], composite face tasks, and morph-based recognition tasks [334–336]. These methodologies could provide more fine-grained insights into the perceptual strategies and neural mechanisms underlying self-face recognition in both autistic and typically developing populations.

### Motor underpinnings of bodily self-representation and their potential as candidate markers in autism

Motor development has increasingly been considered a potentially informative marker of neurodevelopmental differences in ASD [337–339]. Differences in fine and gross motor skills, coordination, gait, and motor planning are frequently reported in autistic individuals and may emerge early in development, sometimes before social and communication characteristics lead to a formal diagnosis [340–342]. However, these motor characteristics may be important not only because of their potential value for early identification, but also because they may reflect broader differences in sensorimotor integration, proprioceptive processing, and action perception coupling [28, 343]. From the perspective of the active self, this relationship is particularly relevant because goal-directed action requires continuous integration of sensory information about the body with predictions about the consequences of movement. Differences in these processes may therefore influence both how actions are organized and how the body is represented and experienced, including aspects of bodily awareness and agency.

The studies reviewed here provide converging, although heterogeneous, evidence that motor differences in autism involve the organization and contextual modulation of action rather than simply reduced motor ability. Kinematic studies in children illustrate this distinction particularly clearly. Chua et al. [194] found that autistic children showed longer movement times and time to peak velocity, lower peak velocity, and a greater number of corrective movement units during goal-directed movements than typically developing children. The developmental pattern of several of these parameters also differed between groups. These findings suggest differences in the balance between prospective feedforward control and subsequent feedback-based correction, with autistic children relying more strongly on corrective processes to achieve the movement goal. Importantly, this pattern does not indicate an inability to perform goal-directed actions, but rather a different strategy for organizing and controlling them.

A complementary pattern was reported by Dowd et al. [195]. Autistic children showed greater trial-to-trial variability in movement preparation, whereas movement execution itself was relatively preserved. Moreover, the presence of a visual distractor altered several aspects of movement planning and execution in typically developing children but produced substantially less modulation in autistic children. Together, the findings of Chua et al. [194] and Dowd et al. [195] suggest that motor differences in autism may arise partly from differences in the prospective use of sensory and contextual information during action planning and online control. This interpretation is consistent with previous evidence of altered motor anticipation and differences in the integration of sensory information during movement planning in autism [124].

The findings of Minissi et al. [196] further demonstrate that these motor differences are sensitive to the specific demands of an action. Using immersive virtual reality, the authors examined whole-body movements during three goal-directed tasks resembling everyday activities. Significant group differences were not observed across all tasks. Instead, differences emerged primarily during the flower-picking task, which required precise and relatively constrained upper-limb movements. During this task, autistic children showed greater displacement of the head, body, and upper limbs, differences in velocity and acceleration, and longer task completion times than typically developing children. The absence of comparable differences during the kicking and bubble tasks indicates that motor atypicalities in autism are not necessarily expressed uniformly across different forms of action. Rather, they may become particularly evident when actions place greater demands on spatial precision, visuomotor coordination, or prospective control. These findings therefore highlight the importance of considering task demands when evaluating motor characteristics as potential markers of autism.

Palmer et al. [197] provided an important link between motor organization and bodily representation. In their rubber hand illusion paradigm, participants with ASD, individuals with high levels of autistic traits, and individuals with low levels of autistic traits showed the expected perceptual effects of synchronous visuotactile stimulation. Subjective illusion ratings, illusion onset latency, and proprioceptive drift did not provide evidence for a general reduction of the perceptual illusion in the ASD group. In contrast, the subsequent influence of the illusion on movement differed according to autistic characteristics. Participants with low levels of autistic traits showed changes in reaching kinematics following synchronous relative to asynchronous stimulation, whereas participants with high autistic traits and those with ASD showed substantially less modulation of their movements by the preceding illusion. Individuals with ASD also showed greater overall movement jerk and differences in the early organization of movement compared with the high autistic-trait group. These findings are particularly relevant because they suggest a dissociation between perceptual embodiment and the use of prior bodily context for subsequent action. Within a predictive processing account, this pattern is compatible with a relatively reduced influence of prior or contextual bodily information during motor control rather than a generalized inability to form an altered bodily representation.

Bertilsson et al. [198] extend these objective findings by emphasizing the subjective experience of body and movement. Young autistic adults reported difficulties in consciously interpreting bodily signals, coordinating movements, maintaining bodily control, and experiencing the body as a coherent whole. Standardized assessments of motor proficiency and movement quality provided complementary information, with the Bruininks Oseretsky Test of Motor Proficiency capturing quantitative aspects of motor performance and the Body Awareness Scale Movement Quality and Experience capturing aspects of movement quality and bodily experience. Importantly, movement quality appeared more closely related to participants’ experiences of everyday functioning than motor quantity alone. These findings suggest that objective motor performance and subjective bodily awareness should not be considered independently. Instead, they may reflect complementary aspects of the sensorimotor processes that contribute to bodily self-representation. Taken together, the reviewed studies suggest that motor characteristics in autism are particularly evident in prospective action organization, contextual modulation of movement, feedback-based correction, visuomotor coordination, and body awareness. The convergence across kinematic, virtual reality, rubber hand illusion, and mixed-methods approaches supports the view that motor function is closely related to broader sensorimotor processes involved in the embodied self. At the same time, the findings do not establish motor measures as autism-specific diagnostic biomarkers. The available evidence instead supports considering kinematic and body-awareness measures as candidate mechanistic or phenotyping markers that may help characterize individual differences in sensorimotor functioning. Their potential diagnostic value will depend on demonstrating that particular motor features are reliable across tasks and developmental stages, predict clinically meaningful outcomes, and show sufficient specificity to distinguish autism from other neurodevelopmental conditions. Motor functioning should therefore not be regarded merely as a peripheral characteristic of autism, but as a potentially informative domain for understanding how sensory information, action organization, and bodily self-representation interact.

#### Motor underpinnings: limitations and future directions

The motor literature is limited by substantial task dependence and by variation in the kinematic variables used to characterize action. Minissi et al. [196], for example, identified group differences during only one of three virtual tasks, whereas Palmer et al. [197] found differences in the influence of prior bodily context on subsequent movement despite broadly comparable perceptual responses to the rubber hand illusion. These findings caution against treating motor differences as a single generalized feature and instead suggest that they may become apparent under particular demands on precision, contextual integration, or prospective control.

Developmental interpretation also requires particular caution. Chua et al. [194] identified age-related differences in movement organization, while Dowd et al. [195] reported greater variability in movement preparation in young autistic children. Cross-sectional findings cannot determine whether such patterns represent delayed maturation, distinct developmental trajectories, compensatory strategies, or stable individual differences. Motor-specific longitudinal studies are therefore needed to establish whether early differences in prospective organization predict later motor function, agency, bodily awareness, or everyday participation.

A further limitation concerns the specificity of the reported motor characteristics. Differences in movement planning, prospective control, sensorimotor integration, coordination, and body awareness are not unique to autism and may also occur in other neurodevelopmental conditions. Consequently, their current value lies primarily in characterizing individual differences in motor and sensorimotor functioning and in identifying candidate mechanistic markers, rather than serving as validated stand-alone diagnostic biomarkers. Future studies should therefore include appropriate neurodevelopmental comparison groups in addition to typically developing controls and should examine the sensitivity and specificity of candidate motor measures before drawing conclusions regarding their diagnostic utility.

Future research would also benefit from greater methodological standardization and from integrating multiple levels of measurement within the same participants. Combining detailed movement kinematics with measures of proprioception, multisensory integration, body ownership, sense of agency, and body awareness could clarify whether the different findings identified across the literature arise from common underlying sensorimotor mechanisms. Such multimethod designs would also help determine whether altered movement organization is associated with differences in bodily self-representation or whether these phenomena can dissociate, as suggested by Palmer et al. [197].

Finally, intervention studies are needed to determine whether motor and body-awareness differences are modifiable and whether changes extend beyond motor performance. The available studies reviewed here do not establish that improving motor prediction, coordination, or body awareness produces changes in agency, self-representation, social functioning, or everyday participation. These possibilities should therefore be treated as testable hypotheses rather than established clinical effects. Controlled longitudinal interventions combining objective motor measures with subjective and functional outcomes could determine whether improvements in sensorimotor functioning generalize to broader aspects of bodily self-representation and daily functioning. Such work would ultimately help establish whether motor atypicalities are primarily associated features of autism or whether they contribute mechanistically to the development and organization of the embodied self.

### Integrating multisensory, self-related, and motor findings

Across the three themes, the reviewed evidence is more consistent with differences in the flexibility and contextual updating of self-related representations than with a global impairment of the bodily self [44, 182, 197]. Relatively preserved performance is reported in several conditions involving basic self-recognition, explicit perceptual judgments, or relatively simple sensory environments [44, 64, 172, 197], whereas differences become more evident when sensory information must be integrated across modalities, when existing bodily representations must be recalibrated, or when prior contextual information must guide ongoing action [44, 84, 168, 170, 182, 197]. Delayed or reduced updating in some bodily-ownership paradigms, differences in multisensory modulation of self-related representations, and reduced context-dependent modulation of motor behavior are broadly consistent with this pattern [44, 75, 84, 168, 170, 197]. Within an active-self framework, these findings suggest that a central question concerns how prior bodily representations, current sensory evidence, and contextual information are dynamically weighted during perception and action. This interpretation remains provisional because few studies directly assessed multiple components of the active self within the same participants.

### Implications for assessment and intervention

The reviewed evidence suggests that multisensory, agency-related, spatial, and motor measures may provide useful information about individual differences in bodily self-processing in autism; however, the current evidence does not support their use as stand-alone diagnostic markers. Measures including bodily-illusion susceptibility and proprioceptive recalibration [84, 168, 170], intentional binding and agency-related measures [96, 179, 180], peripersonal and interpersonal space [149, 176–178, 182], and movement kinematics [194–197] are therefore better regarded at present as mechanistic or phenotyping measures that may complement established clinical assessment. This distinction is particularly important because effects vary across paradigms, developmental stages, and participant characteristics, and several of the candidate processes are not specific to autism.

The evidence also motivates testable intervention hypotheses rather than established treatments. Multisensory training could test whether repeated manipulation of temporal or spatial correspondence alters sensory weighting or the flexibility of bodily and spatial representations [128]. Tool-use paradigms provide another approach for experimentally modifying action-related body and spatial representations, although effects are heterogeneous and require further validation [270–273]. Agency-focused paradigms could examine whether adapting action-effect contingencies modifies perceived control, an idea motivated by evidence linking action-effect timing, agency, and reward-related processes in autism [97, 98]. Motor and body-awareness interventions could similarly test whether changes in prediction, coordination, or proprioceptive awareness generalize beyond motor performance. Demonstrating clinical value will require controlled longitudinal studies, preregistered outcomes, combined objective and subjective measures, and samples representing the broader autism spectrum.

### Cross-cutting strengths, limitations, and future directions

The studies reviewed offer several strengths that lend confidence to the overall pattern of findings. Convergent evidence is drawn from a wide range of methods, including illusion paradigms (rubber hand, numbness, full-body), temporal judgment tasks, measurements of peri- and interpersonal space, agency paradigms, movement kinematics, virtual-reality tasks, and electrophysiology. This methodological breadth supports examining associations across levels ranging from basic sensorimotor integration to higher-order self-other processing and social cognition. A number of designs employ tightly controlled manipulations (e.g., synchrony, temporal delays, spatial congruency, cue weighting), which helps isolate the specific contributions of various sensory modalities. Importantly, some work extends beyond constrained laboratory settings, with virtual-reality and whole-body tasks offering more ecologically relevant assessments of action. The literature spans multiple developmental stages, from childhood to adulthood, and includes a rare longitudinal study of interpersonal space, which begins to address questions of stability and change. Across few areas, effects have been replicated with preregistered analyses, and some studies include cross-diagnostic comparisons. At the same time, clear limitations temper the strength of the inferences. Many samples are small, male-skewed, and predominantly high-functioning, limiting generalizability across the full spectrum, sexes, and cognitive profiles. Protocol heterogeneity persists across laboratories. For example, in the range of visuo-tactile delays, definitions of outcome measures such as proprioceptive drift, and approaches to measuring peri- and interpersonal space. This inconsistency makes quantitative aggregation difficult and potentially adds methodological noise. Most studies are cross-sectional, so developmental trajectories and causal claims remain tentative. Group matching and control of confounds (IQ, attention, sensory reactivity, medication status, comorbidities) are often inconsistent, potentially influencing key outcomes like illusion susceptibility. Several outcome measures depend on subjective reports or behaviors sensitive to demand characteristics, raising questions about construct purity. Although virtual reality improves ecological relevance, many paradigms still rely on artificial stimuli or simplified social contexts, creating a significant ecological gap. Furthermore, not all results converge; preserved performance under unimodal or noiseless conditions suggests boundary conditions that are not yet fully characterized. Finally, mechanistic accounts remain largely correlational; key hypotheses (e.g., predictive processing, altered prior-likelihood balance) are rarely tested with formal computational modeling within the same participants. Taken together, the field is strengthened by its multimethod scope and consistent signals of atypical MSI and self-processing in ASD, yet it would benefit from larger and more diverse samples, greater protocol harmonization, longitudinal and mechanistic designs, and tasks that better approximate everyday action and social contexts. A developmental perspective is particularly important because multisensory processes contributing to body representation continue to mature throughout childhood, with some components of multisensory integration developing into later childhood and adolescence [85, 86, 228, 234]. Some studies included in this review report patterns compatible with delayed visuotactile integration and body-representation updating in autistic children [79, 170, 173], while developmental studies of self-recognition indicate age-related changes in self-processing abilities [187, 188, 284]. At the same time, differences in multisensory and self–other processing and agency-related mechanisms are also reported in autistic adults [44, 179–181], indicating that developmental delay alone is unlikely to explain the full pattern. Because the available literature is overwhelmingly cross-sectional, however, apparent age-related differences should not be interpreted as developmental trajectories. Longitudinal studies following the same individuals across developmental stages are needed to distinguish delayed maturation from persistent differences in multisensory and bodily-self processing.

## Conclusion

This systematic conceptual review indicates that differences in multisensory and sensorimotor processing are associated with several components of bodily self-representation in autism. Across bodily-illusion paradigms, temporal-binding tasks, spatial measures, agency paradigms, self-related tasks, and motor studies, findings indicate differences in multisensory timing and weighting, susceptibility to some bodily illusions, flexibility of peri- and interpersonal boundaries, self-other processing, agency-related mechanisms, and prospective motor organization. However, substantial heterogeneity across developmental stages, participant characteristics, and experimental paradigms argues against a unitary account of the autistic bodily self.

Across the reviewed domains, a recurring pattern is that relatively preserved performance may coexist with differences when bodily or self-related representations must be dynamically updated, reweighted, or adapted to changing multisensory and contextual information. Performance may remain relatively typical under some unimodal, low-noise, or explicit conditions, while differences emerge under conditions requiring multisensory integration, contextual modulation, or prospective action control. This pattern provides a potentially useful framework for linking multisensory processing, self-other differentiation, and action within the active self, but direct mechanistic evidence remains limited.

Future research should prioritize larger and more representative samples, harmonized paradigms, longitudinal and multimethod designs, and direct tests of the proposed mechanisms using psychophysical, kinematic, physiological, electrophysiological, and computational approaches. Multisensory, agency-related, spatial, and motor measures may ultimately contribute to individualized phenotyping or assessment, but their diagnostic specificity and clinical utility remain to be established. Similarly, multisensory, agency-focused, and motor or body-awareness interventions should currently be considered testable research directions rather than established treatments.

## Data Availability

All data can be obtained from the corresponding author (amir.jahanian@uni-bielefeld.de) upon request.

## Abbreviations

BAS MQ-E: Body Awareness Scale – Movement Quality and Experience
ASD: Autism Spectrum Disorder
ASDs: individual diagnosed for autism
TD/TDs: Typically Developing individuals
FBI: Full Body Illusion
AQ: Autism-Spectrum Quotient
QCAE: Questionnaire of Cognitive and Affective Empathy
QCAEcog: QCAE Cognitive Empathy subscale
NI: Numbness Illusion
MA: Mental Age
CA: Chronological Age;
RHI: Rubber Hand Illusion
MIRAGE: name of the multisensory illusion apparatus used to induce spatial visuo-tactile conflict
SAS: Social Aptitude Scale
SZ: Schizophrenia
AS: Asperger Syndrome
HFA/HFAs: High-Functioning Autism/Autistic individuals
PDDNOS: Pervasive Developmental Disorder Not Otherwise Specified
SoA: Sense of Agency
IB: Intentional Binding
OP: Operant condition
CO: Control condition
PDS: Proportion Difference Score
MSI: Multisensory Integration
EEG: Electroencephalography
ERP/ERPs: Event-Related Potential(s)
SPE: Self-Prioritization Effect
FPE: Friend-Prioritization Effect
PDS: Personal Distance Scale
PSC: Private Self-Consciousness Scale
OBT: Own-Body-Transformation
DCD: Developmental Coordination Disorder
TW: Tightrope Walker paradigm
VPT: Visual Perspective Taking
ID: Intellectual Disability
DSR: Delayed Self-Recognition
